# Preparation of trimetallic electrocatalysts by one-step co-electrodeposition and efficient CO_2_ reduction to ethylene†

**DOI:** 10.1039/d1sc06964k

**Published:** 2022-06-10

**Authors:** Shuaiqiang Jia, Qinggong Zhu, Haihong Wu, Shitao Han, Mengen Chu, Jianxin Zhai, Xueqing Xing, Wei Xia, Mingyuan He, Buxing Han

**Affiliations:** Shanghai Key Laboratory of Green Chemistry and Chemical Processes, School of Chemistry and Molecular Engineering, East China Normal University Shanghai 200062 China hhwu@chem.ecnu.edu.cn hanbx@iccas.ac.cn; Beijing National Laboratory for Molecular Sciences, CAS Key Laboratory of Colloid and Interface and Thermodynamics, CAS Research/Education Center for Excellence in Molecular Sciences, Institute of Chemistry, Chinese Academy of Sciences Beijing 100190 China qgzhu@iccas.ac.cn; Institute of Eco-Chongming 20 Cuiniao Road, ChenjiaTown, Chongming District Shanghai 202162 China; Beijing Synchrotron Radiation Facility, Institute of High Energy Physics, Chinese Academy of Sciences Beijing 100049 China

## Abstract

Use of multi-metallic catalysts to enhance reactions is an interesting research area, which has attracted much attention. In this work, we carried out the first work to prepare trimetallic electrocatalysts by a one-step co-electrodeposition process. A series of Cu–X–Y (X and Y denote different metals) catalysts were fabricated using this method. It was found that Cu_10_La_1_Cs_1_ (the content ratio of Cu^2+^, La^3+^, and Cs^+^ in the electrolyte is 10 : 1 : 1 in the deposition process), which had an elemental composition of Cu_10_La_0.16_Cs_0.14_ in the catalyst, formed a composite structure on three dimensional (3D) carbon paper (CP), which showed outstanding performance for CO_2_ electroreduction reaction (CO_2_RR) to produce ethylene (C_2_H_4_). The faradaic efficiency (FE) of C_2_H_4_ could reach 56.9% with a current density of 37.4 mA cm^−2^ in an H-type cell, and the partial current density of C_2_H_4_ was among the highest ones up to date, including those over the catalysts consisting of Cu and noble metals. Moreover, the FE of C_2+_ products (C_2_H_4_, ethanol, and propanol) over the Cu_10_La_1_Cs_1_ catalyst in a flow cell reached 70.5% with a high current density of 486 mA cm^−2^. Experimental and theoretical studies suggested that the doping of La and Cs into Cu could efficiently enhance the reaction efficiency *via* a combination of different effects, such as defects, change of electronic structure, and enhanced charge transfer rate. This work provides a simple method to prepare multi-metallic catalysts and demonstrates a successful example for highly efficient CO_2_RR using non-noble metals.

## Abstract 2

Trimetallic Cu_10_La_1_Cs_1_ catalysts prepared *via* a one-step co-electrodeposition strategy can act as a robust electrocatalyst for CO_2_RR to C_2_H_4_.

## Graphical abstract

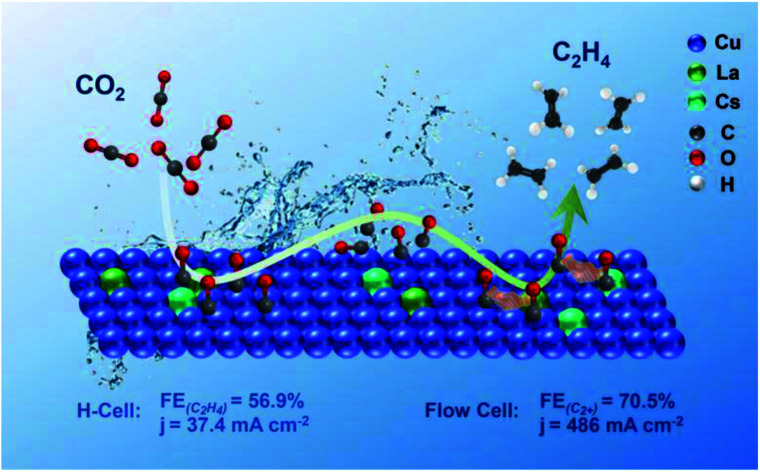

## Introduction

Electrocatalytic reduction of CO_2_ into value-added chemicals is emerging as a sustainable carbon-neutral approach to recycle CO_2_ and store intermittent renewable electricity.^1,2^ As an important C_2_ product of CO_2_RR, C_2_H_4_ is compatible with existing industrial infrastructure and can be used to produce a wide range of chemicals, particularly plastics and polymers.^3–6^ At present, C_2_H_4_ is mainly manufactured by thermal cracking of crude oil-derived naphtha and hydrogenation of CO *via* Fischer–Tropsch synthesis, and the selectivity is generally not high.^7–9^ Although electrocatalytic reduction of CO_2_ provides a straightforward way for C_2_H_4_ production, achieving high selectivity of C_2_H_4_ and high current density remains a challenge. To date, Cu-based catalyst is uniquely active to promote C–C coupling and yield C_2+_ products, but a single Cu catalyst still suffers from unsatisfied selectivity toward specified hydrocarbons.^10–12^ For that, several strategies have been proposed in constructing Cu-based catalysts, including surface reconstruction,^13–15^ hybridization,^9,16^ crystalline faceting,^17,18^ nano/meso-structuring,^19,20^ defect engineering,^21,22^ or creating multi-metallic structural motifs,^23–25^*etc.*

Constructing multi-metallic structures has attracted considerable interest since it can create abundant defects to enhance the CO_2_-to-C_2+_ products activity through optimizing the binding energy among reactants, intermediates, and products with the multi-metallic surface at the nanoscale.^26,27^ So far, the most active doping metallic elements are still noble metals, because the presence of noble metals can easily modulate strains and lattice disorders of the Cu phase, and precisely steer the two pivotal steps towards C_2_H_4_ formation, including *CO formation and C–C coupling.^28,29^ Non-noble metal elements, however, still suffer from low to modest activity. Therefore, the attempts made to conduct efficient CO_2_-to-C_2_H_4_ electrocatalysts rely mostly on bimetallic materials involving noble metals, such as Cu–Au, Cu–Ag, Cu–Pd, *etc.*^30–35^ Unlike the bimetallic catalysts, integrating trimetallic nanostructures manifest great prospects in efficient CO_2_RR to C_2+_ products.^36,37^ To date, however, only Cu–Au/Ag nanoframes have shown promise for enhancing the efficiency of C_2_H_4_ production, in which the CO generation was promoted by the alloyed Ag/Au and the C–C coupling was facilitated by the highly strained and positively charged Cu domains (ESI Table S1†).^36^ However, conventional trimetallic catalysts still require the participation of noble metals and a complicated preparation process. Therefore, the rational design of non-noble trimetallic electrocatalysts with a facial synthesis strategy is highly desired for the practical deployment of electrochemical CO_2_RR.

Herein, we report a one-step strategy to synthesize trimetallic catalysts by co-electrodeposition. A series of trimetallic catalysts Cu–X–Y (X, Y = La, Cs, Zn, Co, Ag, Au) have been developed for CO_2_RR. It was discovered that co-electrodeposition can form a trimetallic composite structure that grown on CP. The presence of non-noble metals La and Cs could create abundant defects and modulate the electronic structure of the Cu phase, offering substantial active sites to stabilize the intermediates and promote C–C coupling to product C_2_H_4_. The as-synthesized trimetallic catalysts also provided large electrochemical surface area and facilitated charge transfer, which enhanced the reaction rate. The high CO_2_ electrocatalytic performance was demonstrated over Cu_10_La_1_Cs_1_ (the concentrations ratio of Cu^2+^, La^3+^, and Cs^+^ in the electrolyte is 10 : 1 : 1 in the deposition process) with up to 56.9% C_2_H_4_ selectivity with a current density of 37.4 mA cm^−2^ in an H-type cell, and a total C_2+_ selectivity of 70.5% with a current density of 485.5 mA cm^−2^ in a flow cell, respectively.

## Results and discussion

**Fig. 1:**
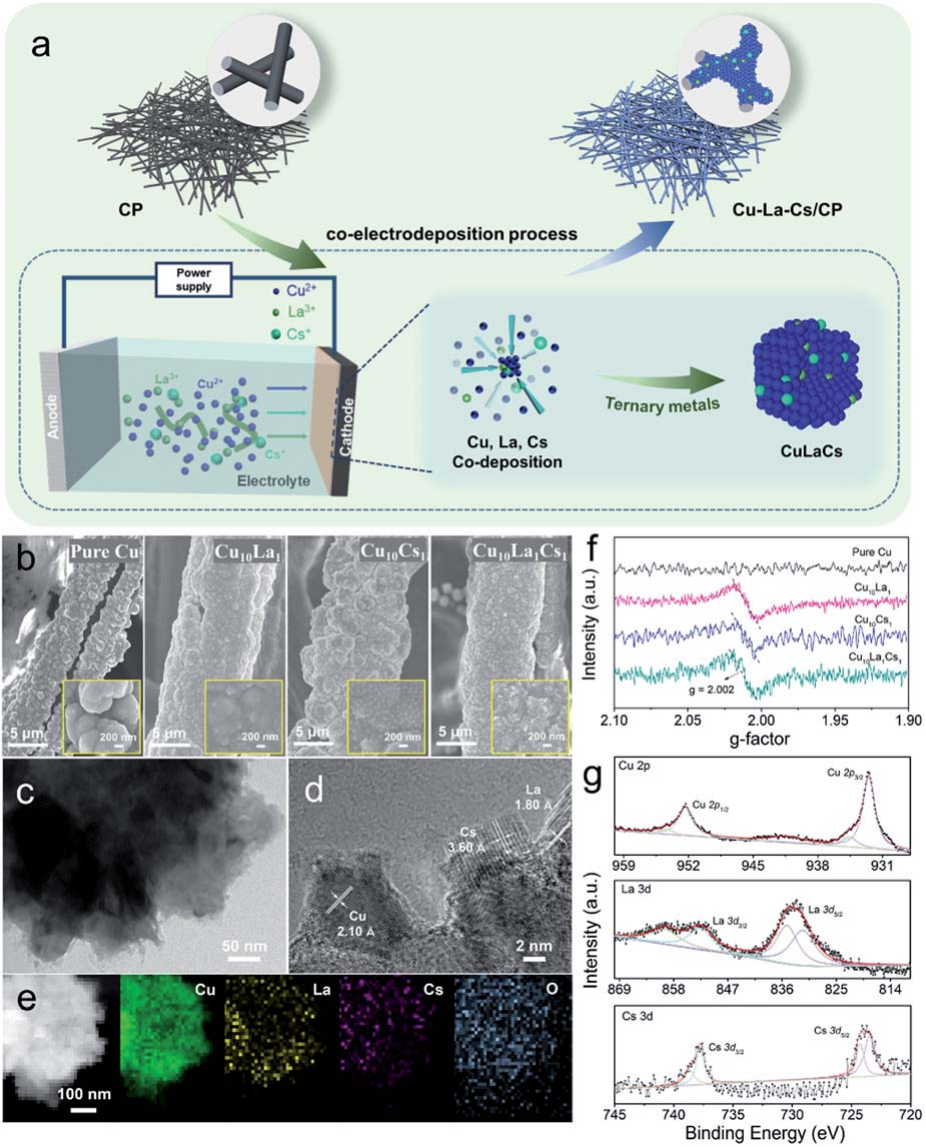
(a) Schematic illustration of the process to prepare pure Cu, bimetallic Cu–La or Cu–Cs, and trimetallic Cu–La–Cs catalysts; structural characterization of Cu–La–Cs catalysts: (b) SEM images of pure Cu, Cu_10_La_1_, Cu_10_Cs_1_, and Cu_10_La_1_Cs_1_ catalysts on a fiber of CP obtained after electrodeposition at a constant voltage of 4 V for 2 min (inset: high-magnification); (c, d) TEM images and HR-TEM image of Cu_10_La_1_Cs_1_ catalyst; and (e) elemental mappings images of Cu_10_La_1_Cs_1_ catalysts; (f) EPR spectra of pure Cu, Cu_10_La_1_, Cu_10_Cs_1_, and Cu_10_La_1_Cs_1_ catalysts at room temperature; (g) XPS spectra of Cu 2p spectra, La 3d spectra, and Cs 3d spectra in Cu_10_La_1_Cs_1_ catalyst.

To understand how trimetallic cooperation might tune the electrocatalytic activity, we prepared a group of trimetallic electrocatalysts (Cu–X–Y; X, Y = La, Cs, Zn, Co, Ag, Au). The non-noble trimetallic catalysts were grown on three-dimension CP (ESI Fig. S1†) through a one-step co-electroplating process, which was illustrated in Fig. 1a using trimetallic Cu–La–Cs as a representative example. Typically, a piece of CP with a geometric area of 1 cm^2^ and a Pt gauze were used for the cathodic and anodic electrodes with a gap of 1 cm, and the electrochemical experiments could be controlled by a DC Power supply. Before all the experiments, the CP was ultrasonically cleaned with acetone, ethanol, and deionized water. For the Cu_10_La_1_Cs_1_ electrode, the electrodeposition was carried out cathodically using a 50 mL solution of H_2_SO_4_ (10 mM), Cu(ii) gluconate (100 mM), La(iii) acetate (10 mM), Cs(i) acetate (10 mM), and 4-aminopyridine (10 mM). The deposition was carried out at a constant voltage of 4 V for 2 min. When the molar ratio of Cu(ii), La(iii), and Cs(i) in the electrolyte was 10 : 1 : 1, the as-synthesized catalyst was denoted as Cu_10_La_1_Cs_1_ with a total loading of 1.33 mg metals on 1.0 cm^−2^ CP (Table S2†), and the contents of Cu, La, and Cs in Cu_10_La_1_Cs_1_ were 93.92 wt%, 3.31 wt%, and 2.77 wt% (Table S3†), respectively, as determined by inductively coupled plasma optical emission spectroscopy (ICP-OES).

The scanning electron microscopy (SEM) images reveal that trimetallic Cu_10_La_1_Cs_1_ film grew uniformly on the fiber of the 3D CP substrate (Fig. 1b). Inset in Fig. 1b shows that the Cu, Cu_10_La_1_, Cu_10_Cs_1_, and Cu_10_La_1_Cs_1_ catalyst films had a rough surface. Besides, transmission electron microscopy (TEM) images confirm the formation of Cu_10_La_1_Cs_1_ trimetallic nanostructure, which was different from the pure Cu structure (Fig. 1c, d and S2†).

The high-resolution TEM (HR-TEM) image exhibit that the interplanar spacing was 2.10 Å, corresponding to the d spacing of (111) plane of Cu (Fig. 1d and S3a, b†), which is similar to pure Cu catalyst (Fig. S2†). In addition, the observed fringes with an interlayer spacing of 3.60 Å and 1.80 Å correspond to the (012) plane of Cs_2_O (JCPDS card no. 09-0104), and the (211) plane of La_2_O_3_ (JCPDS card no. 40-1284) (Fig. 1d and S3c–f†). The elemental distribution mapping (EDS) further confirmed the uniform dispersion of Cu, La, and Cs species in the trimetallic Cu_10_La_1_Cs_1_ catalysts (Fig. 1e). The abundant vacancies and lattice disorder could also be observed in Cu_10_La_1_Cs_1_ (Fig. S4 and S5†), which indicates that defect-rich nanostructure existed in the catalyst. Such defects result in high exposure of coordination-unsaturated Cu sites, which may change the electronic structure of Cu and influence its catalytic performance.^38,39^ The electron paramagnetic resonance (EPR) spectra of different samples were collected at room temperature (Fig. 1f). Compared with pure Cu, typical signals for oxygen vacancy appeared at *g*-value of 2.002 for Cu_10_La_1_, Cu_10_Cs_1_, and Cu_10_La_1_Cs_1_ catalysts, which indicates that introducing of La or Cs contributes to the formation of defects (Fig. 1f). The oxygen vacancy was also reported to optimize the adsorption energy of reactants on the catalyst surface, which reduces the reaction energy barrier and promotes molecular activation.^40^

The time-dependent X-ray diffraction (XRD) showed the gradual formation of trimetallic Cu_10_La_1_Cs_1_ catalysts (Fig. S6†). In detail, the representative peaks could be observed at 43.2°, 50.4°, 74.1°, 89.9°, and 37.0°, which can be indexed to the Cu (111), Cu (200), Cu (220), Cu (311), and Cu_2_O (111) planes. However, the diffraction peaks of La and Cs were not shown obviously for trimetallic Cu_10_La_1_Cs_1_, because their amounts were below the XRD detection threshold. The X-ray photoelectron spectroscopy (XPS) and X-ray absorption spectroscopy (XAS) analyses were performed to further investigate the surface chemical composition and elemental valence states of the catalyst. XPS results showed that the surface of Cu_10_La_1_Cs_1_ was composed of Cu, La, and Cs species (Fig. 1g and S7†). The peaks at 932.1 eV (Cu 2p_3/2_) and 952.0 eV (Cu 2p_1/2_) retained the characteristic feature of Cu species, which was further confirmed by the Cu 2p and Auger Cu LMM spectra that the Cu existed as Cu^0^ and Cu^+^, and Cu^0^ was predominant (Fig. S8†). In addition, the XPS spectra could also be fitted to include both La(iii) and Cs(i) species, which correspond to peaks at 835.6 eV (La 3d_5/2_) and 852.5 eV (La 3d_3/2_), 723.9 eV (Cs 3d_5/2_) and 743.1 eV (Cs 3d_3/2_), respectively (Fig. 1g). These results showed that the Cu, La, and Cs coexisted in the Cu_10_La_1_Cs_1_ catalysts. To further study the electronic structures and chemical bonding of the Cu phase that was influenced by La and Cs atoms, we performed X-ray absorption spectroscopy (XAS) analysis. The Cu K-edge X-ray absorption near-edge spectra (XANES) spectra of Cu_10_La_1_Cs_1_ with the reference materials indicated that Cu_10_La_1_Cs_1_ were mainly composed of Cu^0^ and Cu^+^. From the XANES spectrum, it is obvious that the dominated Cu–Cu coordination at 2.23 Å existed in the catalyst, which is identical to that of Cu^0^ (Fig. S9†). This is in good agreement with the XRD and XPS analyses.

**Fig. 2:**
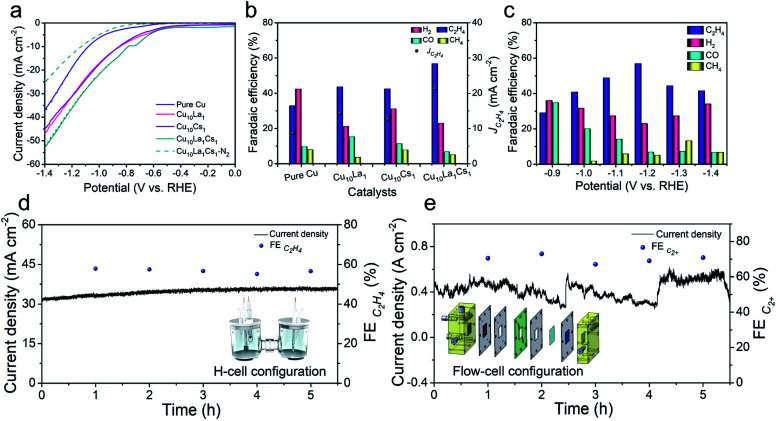
(a) LSV traces at a scan rate of 50 mV s^−1^ of pure Cu, Cu_10_La_1_, Cu_10_Cs_1,_ and Cu_10_La_1_Cs_1_ catalysts in CO_2_-saturated and N_2_-saturated 0.1 M KCl aqueous electrolyte; (b) the distribution of reduction products and partial current densities for C_2_H_4_ at −1.2 V *vs.* RHE over pure Cu, Cu_10_La_1_, Cu_10_Cs_1,_ and Cu_10_La_1_Cs_1_ catalysts; (c) the distribution of reduction products at different applied potentials over Cu_10_La_1_Cs_1_ catalysts; (d) electrochemical stability test of Cu_10_La_1_Cs_1_ film electrode at −1.2 V *vs.* RHE in an H-type cell; (e) electrochemical stability test of Cu_10_La_1_Cs_1_ film electrode at −0.97 V *vs.* RHE in a flow cell.

The as-synthesized catalysts were firstly tested for CO_2_RR in 0.1 M KCl aqueous electrolyte using a typical H-type cell. The low buffering capability of KCl aqueous electrolytes allowed the electrode surface pH to increase to a weakly basic range, which facilitated the formation of C_2_ products by combining with efficient electrocatalysts.^41,42^ In this study, the linear scanning voltammetry (LSV) curves over various catalysts were determined, including pure Cu, bimetallic Cu_10_La_1_, Cu_10_Cs_1_, and trimetallic Cu_10_La_1_Cs_1_ catalysts. As shown in Fig. 2a, over Cu_10_La_1_Cs_1_ catalyst, the current density (*j*) was much higher in CO_2_-saturated electrolyte than that in N_2_-saturated electrolyte from −0.5 V to −1.4 V *vs.* RHE, suggesting the reduction of CO_2_. Moreover, in CO_2_-saturated electrolyte, the current density over Cu_10_La_1_Cs_1_ was higher than that over other catalysts. The electrolysis performances at different potentials over various catalysts are displayed in Fig. 2b, c, and S10–S12.† Under the reaction conditions, the gaseous products were mainly composed of H_2_, CO, CH_4_, and C_2_H_4_, which were determined using gas chromatography (GC). The liquid products were evaluated by nuclear magnetic resonance (NMR), and only very small amounts of formic acid (<1%) were detected. The content was very low and was not considerable (Fig. S14†). Clearly, Cu_10_La_1_Cs_1_ had a much higher FE of C_2_H_4_ than other catalysts at all potentials (Fig. 2b). For pure Cu catalyst, the FE of C_2_H_4_ was only 33.0% at −1.2 V *vs.* RHE, which exhibited an inferior catalytic performance compared with other catalysts (Fig. 2b). For bimetallic Cu_10_La_1_ and Cu_10_Cs_1_, the FE of C_2_H_4_ increased consistently to 43.6% and 42.5% respectively. The FE of C_2_H_4_ over trimetallic Cu_10_La_1_Cs_1_ catalyst could reach 56.9% with a current density of 37.4 mA cm^−2^ at −1.2 V *vs.* RHE, suggesting a 2.7-fold increase in partial current density, compared to the pure Cu catalyst (Fig. 2b and S10–S12†). We also synthesized trimetallic Cu–La–Cs catalysts with different Cu–La–Cs ratios. Clearly, Cu_10_La_1_Cs_1_ had the highest catalytic CO_2_RR activity toward C_2_H_4_ production (Fig. S13†). Systematic comparisons to state-of-the-art catalysts revealed that the performance of the trimetallic Cu_10_La_1_Cs_1_ catalyst with rich-defects was one of the best ones in the H-type cell (Table S1†), including those over the catalysts consisting of Cu and noble metals. Long-term electrolysis was also performed to verify the stability of the catalyst. As shown in Fig. 2d, the current density and FE of C_2_H_4_ over Cu_10_La_1_Cs_1_ were not changed obviously at −1.2 V *vs.* RHE for 5 h, indicating the stability of the catalyst at the CO_2_RR condition. After the reaction, the XRD, EPR, and XPS analyses were performed and the results showed that the properties of the catalyst did not change noticeably (Fig. S15–S17†). The EXAFS at the Cu K-edge confirmed a well-retained Cu interaction in samples collected after the CO_2_RR test (Fig. S18†). These results also indicated the remarkable stability of the trimetallic Cu_10_La_1_Cs_1_ catalyst.

In addition, to gauge the benefits of the trimetallic Cu–La–Cs catalyst for high-rate CO_2_RR, we translated the catalyst to a gas-diffusion environment (Fig. S19†). In this configuration, hydrophobic CP containing carbon black layer acted as a gas diffusion electrode (GDE). When using 1 M KOH as the electrolyte, we found that Cu_10_La_1_Cs_1_ could maintain C_2+_ selectivity up to 70.5% with a high current density of 485.5 mA cm^−2^ at a low reduction potential of −0.97 V *vs.* RHE. The FE of C_2+_ products were ethylene (42.1%), ethanol (20.8%), and *n*-propanol (7.6%), respectively (Fig. S20†). The configuration also showed a stable potential profile over 5 h without noticeable decay of the current density and C_2+_ product selectivity (Fig. 2e).

Considering the experimental observations above, we think that the excellent performance of the Cu_10_La_1_Cs_1_ catalyst resulted partially from the synergistic effect of the components in the trimetallic catalyst. We found that the surface roughness of the catalysts changed obviously with the introduction of La and Cs, which is beneficial for the increasing of active sites.^43^ The values of electrochemical double-layer capacitance (*C*_dl_) were calculated from cyclic voltammograms (CV) curves (Fig. S21†) to evaluate the electrochemical active surface area (ECSA) of different catalysts. The *C*_dl_ value of Cu_10_La_1_Cs_1_ film was calculated to be 4.51 mF cm^−2^, which was obviously higher than that of others (Fig. S22†). It suggested that Cu_10_La_1_Cs_1_ film with rich-defects could provide more catalytic sites, leading to an increase in reaction rate during the electrocatalytic process. We also used electrochemical impedance spectroscopy (EIS) to study the interfacial properties of the catalysts at an open circuit potential in a CO_2_-saturated electrolyte (Fig. S23†). The Cu_10_La_1_Cs_1_ catalyst showed the lowest charge transfer resistance (*R*_ct_). Therefore, the charge transport is more facile on Cu_10_La_1_Cs_1_ catalyst, which is favorable to enhance the reaction rate.

**Fig. 3:**
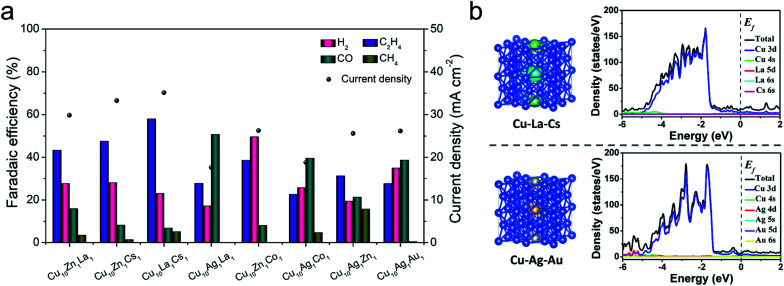
(a) The distribution of reduction products and current density at −1.2 V *vs.* RHE over different metal catalysts; (b) the structure models and the DOS of Cu_10_La_1_Cs_1_ and Cu_10_Ag_1_Au_1_ electrodes. (The atoms in blue, green, viridian, silvery, and brown represent Cu, La, Cs, Ag, and Au, respectively. The doping model shown is Cu 2 × 2 × 2 supercell-based substitution doping).

To screen the synergistic effect of the components in the trimetallic catalysts, we also prepared a series of Cu_10_–X_1_–Y_1_ catalysts using the same method. When the molar ratio of metal ions for Cu, X, and Y in the electrodeposition electrolyte was 10 : 1 : 1, the as-synthesized catalyst was denoted as Cu_10_X_1_Y_1_. The compositions of these trimetallic catalysts, such as Cu_10_Ag_1_La_1_, Cu_10_Zn_1_La_1_, Cu_10_Ag_1_Cs_1_, Cu_10_Zn_1_Co_1_, and Cu_10_Ag_1_Au_1_, were then characterized by ICP-OES, and their element compositions are shown in Tables S3 and S4.† It indicates that the elemental compositions of the trimetallic catalysts varied with different doping components. The CO_2_RR performance tests of different trimetallic catalysts were conducted, and the results are shown in Fig. 3a. It indicates that the catalytic performances of the metals depended on the metals introduced into Cu. Some metal doping (*e.g.* Ag, Co, and Au) can promote the production of C_1_ products, while other metal doping (*e.g.* Zn, La, and Cs) can inhibit hydrogen evolution and promote C–C coupling. Among them, Cu_10_La_1_Cs_1_ yielded the highest FE of C_2_H_4_ and current density. The multi-metallic system could actively generate CO or inhibit cathodic hydrogen evolution reaction during the electrochemical reduction of CO_2_. The excess amount of CO molecules on the metal surface are expected to migrate to the adjacent Cu surface and then undergo C–C coupling for the formation of C_2_H_4_ products.^6,30,44^ The study indicated that the compositions not only significantly influenced the surface morphology of the trimetallic structures (Fig. S24†), but also changed the electronic properties of the catalyst. We then compare the effect of different defect/vacancies on the electronic properties, using Cu_10_La_1_Cs_1_ and Cu_10_Ag_1_Au_1_ as a comparing couple. The calculated density of states (DOS) of Cu_10_La_1_Cs_1_ and Cu_10_Ag_1_Au_1_ are shown in Fig. 3b. Comparing with the partial density of states (PDOS) for Cu_d_ orbitals, the electronic environment (the gap states) of the catalyst is constructed jointly by all the metals after doping with La/Cs or Ag/Au, which suggests that doping can significantly promote the electron transfer of the catalyst.^45,46^ It suggests that La/Cs doping has a similar effect as Ag/Au doping in regulating the electronic structure of Cu-based catalyst, which is beneficial to the charge transfer for CO dimerization.^47–49^ Therefore, we can conclude that the synergistic effect of the components in the trimetallic catalysts can be attributed to the change of different defects/vacancies on the electronic properties and surface structures.

**Fig. 4:**
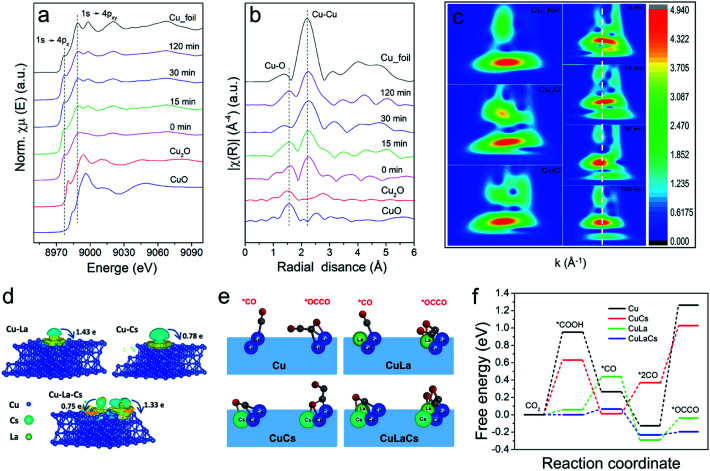
Semi-in situ XAS characterization and DFT calculations. (a) Normalized Cu K edge XANES spectra of Cu_10_La_1_Cs_1_ during CO_2_RR at −1.2 V *vs.* RHE; (b) corresponding *k*^3^-weighted FT-EXAFS spectra of Cu_10_La_1_Cs_1_ during CO_2_RR at −1.2 V *vs.* RHE; (c) Morlet WT of the *k*^3^-weighted EXAFS data for Cu_10_La_1_Cs_1_ during CO_2_RR at −1.2 V *vs.* RHE; (d) side views of the charge density difference of Cu_10_La_1_, Cu_10_Cs_1_, and Cu_10_La_1_Cs_1_ with an isosurface of 6 × 10^−4^, 2 × 10^−3^ and 2 × 10^−3^ e Å^−3^, respectively. (The charge accumulation is shown as the yellow region, and the charge depletion is shown as the cyan region); (e) the *CO and *OCCO adsorption configurations on pure Cu, Cu_10_La_1_, Cu_10_Cs_1,_ and Cu_10_La_1_Cs_1_ interface structures; (f) the activation energy barrier of CO dimerization at different models.

It is worth mentioning that no obvious changes occurred in Cu phase during CO_2_RR. From the semi-in situ XAS characterization results presented in Fig. 4a–c, we can find that the surface of Cu_10_La_1_Cs_1_ was still mainly Cu^0^ sites with the increase of electrolysis time. These data further confirm that the La and Cs components could maintain the chemical state and the local coordination environment of the Cu phase was not changed under reaction conditions. The undercoordinated Cu sites are associated with outstanding C–C coupling.^50–52^ We then pursued theoretical insights into the study of the intrinsic property of the catalysts. As depicted in computational structure models in Fig. 4d and S25–S30,† the interactions and electronic structure among Cu, La, and Cs atoms of pure Cu, Cu_10_La_1_, Cu_10_Cs_1_, and Cu_10_La_1_Cs_1_ catalysts were different. For Cu_10_La_1_Cs_1_, the La and Cs atoms tend to delocalize charge by releasing electrons to the Cu atoms, manifesting the electron transfer effect. The optimized adsorption configurations of reaction intermediates on the simulated interface structures are displayed in Fig. S31.†Fig. 4e and f show the *CO and *OCCO adsorption configurations on the four simulated interface structures. We can find that the presence of La and Cs in Cu_10_La_1_Cs_1_ could effectively adjust the adsorption space position of *CO and *OCCO intermediates to an optimized state, which enables the lowest energy barrier for CO_2_ transformation to more reduced products with the multi-electron process. This result is obviously different from that over pure Cu catalyst, on which the energy barrier is mainly in the typical two steps of CO_2_ hydrogenation reduction to generate adsorbed carboxylic acid groups (CO_2_ + H^+^ + e^−^ → *COOH) and CO molecular copolymerization (*2CO → *OCCO), requiring the high energy barrier.^53^ With the introduction of the Cs atom, the energy barrier of these two steps was reduced, and it becomes more noticeable with the further introduction of La atoms. Therefore, we consider that the synergistic effect between Cu, La, and Cs not only reduces the energy barrier for the CO_2_ hydrogenation reduction to form adsorbed carboxylic acid groups (CO_2_ + H^+^ + e^−^ → *COOH) and CO molecules (*COOH + H^+^ + e^−^ + H_2_O → *CO), but also promotes the C–C coupling process for C_2_H_4_ formation (*2CO → *OCCO).^54–57^ This suggests that the presence of La and Cs favors CO production and leads to higher CO* coverage around the active sites. The increased CO* coverage then improves the dimerization of neighboring *CO intermediates to generate *OCCO rather than desorbed. The above results, taken together, suggest that the synergistic interaction among Cu, La, and Cs can efficiently enhance the C_2_H_4_ selectivity *via* a combination of effects, including defects, change of electronic structure, fast charge transfer rate, and increase of active sites.

## Conclusions

In summary, we find that a trimetallic catalyst prepared *via* a one-step co-electrodeposition strategy can act as a robust electrocatalyst for CO_2_RR to C_2_H_4_. In particular, over Cu_10_La_1_Cs_1_ catalyst, the C_2_H_4_ selectivity can reach 56.9% with a current density of 37.4 mA cm^−2^ in an H-type cell, and a total C_2+_ selectivity reaches 70.5% with a current density of 485.5 mA cm^−2^ in a flow cell, respectively. The outstanding electrocatalytic performance of the trimetallic Cu_10_La_1_Cs_1_ catalyst can be ascribed to the synergistic effect of Cu, La, and Cs. The abundant defects can modulate the electronic structure of the Cu phase, offering substantial potential active sites to stabilize the *CO intermediates and promote C–C coupling to produce C_2_H_4_. The as-synthesized trimetallic catalysts on 3D CP also result in a large electrochemical surface area and fast charge transfer, which enhance the reaction rate. We believe that the methodology to prepare multi-metallic catalysts by co-electrodeposition can also be used to design other efficient catalysts for CO_2_RR.

## Data availability

The authors declare that all data supporting the findings of this study are available within the paper [and its ESI†].

## Author contributions

S. Q. J., Q. G. Z., H. H. W. and B. X. H. proposed the project, designed the experiments, and wrote the manuscript. S. Q. J. performed the whole experiment. S. T. H., M. E. C., J. X. Z., X. Q. X. and W. X. performed the analysis of experimental data. Q. G. Z., H. H. W., M. Y. H. and B. X. H. co-supervised the whole project. All authors discussed the results and commented on the manuscript.

## Conflicts of interest

The authors declare no competing interests.

## Supplementary Material

SC-013-D1SC06964K-s001
