# Supplementary material for: Preparation of trimetallic electrocatalysts by one-step co-electrodeposition and efficient CO2 reduction to ethylene
Source: Chem Sci. 2022 Jun 10;13(25):7509–15. doi: 10.1039/d1sc06964k (PMC9241956; doi:10.1039/d1sc06964k)
Supplement: SC-013-D1SC06964K-s001 [file SC-013-D1SC06964K-s001.pdf]

Electronic Supplementary Material (ESI) for Chemical Science.
This journal is © The Royal Society of Chemistry 2022

Supporting Information

Preparation of trimetallic electrocatalysts by one-step co-electrodeposition and efficient CO_2 reduction to ethylene

Shuaiqiang Jia,[a, c] Qinggong Zhu,*[b] Haihong Wu,*[a, c] Shitao Han,[a, c] Mengen Chu,[a, c] Jianxin Zhai,[a, c] Xueqing Xing,[d] Wei Xia,[a, c] Mingyuan He,[a, c] and Buxing Han*[a, b, c]

[a] Shanghai Key Laboratory of Green Chemistry and Chemical Processes, School of Chemistry and Molecular Engineering, East China Normal University, Shanghai 200062, China.

[b] Beijing National Laboratory for Molecular Sciences, CAS Key Laboratory of Colloid and Interface and Thermodynamics, CAS Research/Education Center for Excellence in Molecular Sciences, Institute of Chemistry, Chinese Academy of Sciences, Beijing 100190, China.

[c] Institute of Eco-Chongming, 20 Cuiniao Road, ChenjiaTown, Chongming District, Shanghai 202162, China.

[d] Beijing Synchrotron Radiation Facility, Institute of High Energy Physics, Chinese Academy of Sciences, Beijing 100049, China.

Materials

Copper(II) gluconate (min, 98%) was obtained from Strem Chemicals, Inc. Sodium borohydride (98%), methanol, ethanol, acetone, potassium sulphate, potassium chloride, hydrochloric acid (36-38%), and sulfuric acid (95-98%) were provided by Sinopharm Chemical Reagent Co., Ltd. Lanthanum(III) acetate, cesium(I) acetate, cobalt(II) acetate, zinc acetate, 3,5-Diamino-1,2,4-triazole (DAT, 98%), cupric acetate, copper (Cu) standard solution, lanthanum (La) standard solution and cesium (Cs) standard solution were purchased from Shanghai Aladdin Bio-Chem Technology Co., LTD. Polytetrafluoroethylene (PTFE, 60 wt%), silver acetate, lanthanum chloride, 4-aminopyridine, tetrachloroauric(III) acid trihydrate (99.9%, Au 50%), and ammonium persulphate (99.99%) were obtained from Beijing Innochem Science & Technology Co., Ltd. Toray Carbon Paper (CP, TGP-H-60, 19×19 cm), Nafion N-117 membrane (0.180 mm thick, ≥0.90 meg/g exchange capacity) and Nafion D-521 dispersion (5% w/w in water and 1-propanol, ≥ 0.92 meg/g exchange capacity) were purchased from Alfa Aesar China Co., Ltd. Both CO_2 and N_2 had a purity of 99.999 %, which were provided by Shanghai Chemistry Industrial Zone Pujiang Special Type Gas Co., Ltd. All the chemical reagents were used directly without further purification.

Catalyst Preparation

Electrosynthesis of pure Cu catalyst: Typically, a piece of CP with a geometric area of 1 cm^2 and a platinum gauze were used for the cathodic and anodic electrodes with a gap of 1 cm, and the electrochemical experiments could be controlled by a DC Power supply (Hangzhou Huayi Electronics Industry Co., Ltd.). Before all the experiments, the CP was ultrasonically cleaned with acetone, ethanol, and deionized water. The electrodeposition was carried out cathodically using 50 mL solution of H_2SO_4 (10 mM), Copper(II) gluconate (100 mM), and 4-aminopyridine (10 mM). The deposition was carried out at a constant voltage of 4 V for desired time. Then the as-prepared electrode was washed with water and ethanol several times and dried at room temperature in a vacuum oven before use.

Electrosynthesis of $Cu_{10}La_1Cs_1$, $Cu_{10}La_1$, and $Cu_{10}Cs_1$ catalysts: Typically, a piece of CP with a geometric area of 1 cm^2 and a platinum gauze were used for the cathodic and anodic electrodes with a gap of 1 cm, and the electrochemical experiments could be controlled by a DC Power supply. Before all the experiments, the CP was ultrasonically cleaned with acetone, ethanol, and deionized water. For the $Cu_{10}La_1Cs_1$ electrode, the electrodeposition was carried out cathodically using 50 mL

solution of H_2SO_4 (10 mM) , Copper(II) gluconate (100 mM), Lanthanum(III) acetate (10 mM), cesium acetate (10 mM), and 4-aminopyridine (10 mM). The deposition was carried out at a constant voltage of 4 V for desired time. Then the as-prepared electrode was washed with water and ethanol several times and dried at room temperature in a vacuum oven before use. $Cu_{10}La_1$ and $Cu_{10}Cs_1$ electrodes were prepared by this method with different combinations of metal salts mixed solution.

By using this method, Cu-X-Y with different metals (e.g. La, Cs, Zn, Co, Ag, Au), could be prepared.

Electrosynthesis of $Cu_{10}La_1Cs_1$ based GDEs: The preparation of $Cu_{10}La_1Cs_1$ based GDEs were similar to that to fabricate the $Cu_{10}La_1Cs_1$ electrode except that CP was replaced with a hydrophobic carbon paper containing a carbon black layer. After washing and drying, the sample was immersed into PTFE dispersion (10 wt %) for a few seconds to adsorb PTFE into the pores.

Characterization

X-ray diffraction patterns were acquired by an X-ray diffractometer (XRD; Rigaku Ultima VI X–ray) with Cu-Kα radiation (λ=1.54 Å). The morphologies of the samples were observed by field-emission scanning electron microscopy (SEM) (Hitachi S4800) and transmission electron microscope equipped with EDS (TEM, JEM-2100F) operated at 200 kV. The valence states and composition of the samples were examined by X-ray photoelectron spectroscopy (XPS) on an AXIS Supra surface analysis instrument using a monochromatic Al Kα X-ray beam (1,486.6 eV). Before the XPS measurements, the catalysts were stored under ambient conditions. This effectively preserved the oxidation of the samples during the sample transfer. The content of metals in the catalysts was determined by inductively coupled plasma optical emission spectroscopy (ICP-OES, Optima 8300, Perkin-Elmer). Comparison to Cu, La, and Cs standards of known concentration allowed the determination of the respective Cu, La, and Cs contents. Electronic paramagnetic resonance (EPR) spectra were recorded at room temperature, in continuous-wave mode, on Bruker EMXplus-10/12 spectrometers, at mw power of ~0.63 mW and modulation amplitude 10 G; spectra reported herein were typically obtained over an average of 20 scans. The X-ray absorption spectroscopy (XAS) experiments were carried out at the 4B9A beamline at Beijing Synchrotron Radiation Facility (BSRF), China. Data analysis of Cu K-edge X-ray absorption near-edge spectroscopy (XANES) and extended X-ray absorption fine structure (EXAFS) spectra were conducted using the Athena software package. Pre-edge and post-edge backgrounds were subtracted from the XAS spectra, and the resulting spectra were normalized by edge height.[1]

Electrochemical study

A gas-tight two-compartment H-cell separated by a proton exchange membrane (Nafion117) and a CHI 660E potentiostat workstation (Shanghai CH Instruments Co., China) were used, and the experiment was conducted at room temperature. The anode and cathode sides were filled with 30 mL of 0.1 M K_2SO_4 and 0.1 M KCl, respectively. Before each set experiment, the catholyte was bubbled with CO_2 or N_2 for at least 30 min to form a CO_2-saturated or N_2-saturated solution. Linear sweep voltammetric (LSV) scans were conducted in an H-type cell with a three-electrode configuration, which consisted of a working electrode, a platinum gauze counter electrode, and an Ag/AgCl (saturated KCl solution) reference electrode. LSV measurements in gas-saturated electrolytes were carried out in the potential range of 0.2 V to –1.4 V vs. RHE at a scan rate of 20 mV s^{-1}. In all measurements, all electrochemical data were referenced to RHE using equations:

$$E\ (\text{V } vs. \text{ RHE}) = E\ (\text{V } vs. \text{ Ag/AgCl.}) + E^0\ (\text{V of Ag/AgCl. } vs. \text{ NHE}) + 0.059\times\text{pH}$$

Where E^0 (V of Ag/AgCl. *vs.* NHE) = 0.197 V.

Electrolysis in an H-type cell

The as-synthesized electrodes were used as the working electrode. In the experiments, the Nafion-117 membrane was used as a proton exchange membrane to separate the cathode and anode compartments. In the electrolysis experiment, the amount of electrolyte was 30 mL, and the catholyte were bubbled with CO_2 for at least 30 min to form CO_2 saturated solution, and the potentiostat electrochemical reduction was carried out under a steady stream of CO_2 (15 sccm). First, the cathode side was electrochemically reduced using the cyclic voltammetric (CV) method, which ranged from 0.6 to −1.4 V vs. RHE at a rate of 0.1 V s^{-1} for 5 cycles to completely reduce the possible oxidized species.

Electrolysis in a flow cell

The $Cu_{10}La_1Cs_1$ GDEs were evaluated in a flow-cell reactor, which consisted of a gas diffusion chamber, a cathodic counterpart, and an anodic counterpart. CO_2 was introduced to the gas diffusion chamber at 15 mL min^{-1}. 1.0 M KOH electrolyte (70 mL) was circulated (at 20 mL min^{-1}) using a peristaltic pump through the cathodic counterpart, and another 70 mL 1.0 M KOH of electrolyte was circulated through the anodic counterpart.

Products analysis

After electrolysis, the gaseous products were collected and analyzed by gas chromatography (GC, Agilent–8890). From the GC peak areas and calibration curves of the TCD detector, the moles of a gaseous product can be calculated. The liquid products were quantified by a nuclear magnetic resonance (NMR) spectrometer. 1H NMR spectra of freshly acquired samples were collected on an NMR spectrometer (Bruker; Ascend 400–400 MHz) in deuterated water (D_2O) with phenol as an

internal standard.

After the quantification, the FE of each product was calculated as follows:

$$FE = \frac{n_{electrons} \times F \times \text{moles of product}}{Q} \times 100\%$$

(Q: the amount of charge passed through the working electrode; F: The Faraday constant (96485 C mol^{-1}); $n_{electrons}$: the number of electrons transferred for product formation).

Double-layer capacitances (C_{dl}) measurement

The value of C_{dl} is proportional to the electrochemically active surface area. The value of C_{dl} was determined by measuring the capacitive current associated with double-layer charging from the scan-rate dependence of cyclic voltammogram (CV) in an H-type cell. The CV was obtained from −0.1 V to −0.2 V vs. RHE. Scans were recorded at different scan rates with a minimum of 3 cycles in the non-Faradaic region, which included 10 mV s^{-1}, 20 mV s^{-1}, 40 mV s^{-1}, 60 mV s^{-1}, 80 mV s^{-1}, 100 mV s^{-1}, 150 mV s^{-1}, and 200 mV s^{-1}. The C_{dl} was estimated by plotting the Δj ($j_a - j_c$) at –0.15 V vs. RHE (0.1 M KCl solution) against the scan rates, where j_a and j_c were the anodic and cathodic current density, respectively.

Electrochemical impedance spectroscopy (EIS)

The EIS measurements were carried out in CO_2 saturated 0.1 M KCl solution at an equilibrium potential. The impedance spectra were recorded with an amplitude of 5 mV of 10^{-2} to 10^5 Hz. The data obtained from the EIS measurements were fitted by the Zview software (Version 3.1, Scribner Associates, USA).

Computational Method

DFT calculations were conducted through the Vienna ab initio Simulation Package (VASP) with the projector augmented wave method.[2, 3] Generalized gradient approximation of the Perdew-Burke-Ernzerhof (PBE) functional was used as the exchange-correlation functional.[4, 5] The cutoff energy was set as 500 eV, and structure relaxation was performed until the convergence criteria of energy and force reached 1×10^{-5} eV and 0.02 eV Å^{-1}, respectively. The Brillouin zone was sampled with 3 × 3 × 3 K points for bulk optimization and 2 × 2 × 1 K points for surface calculation. A vacuum layer of 15 Å was constructed to eliminate interactions between periodic structures of surface models. For simulating an actual surface condition, the bottom two layers of slabs were fixed, and the top two layers were allowed to relax. The van der Waals (vdW) interaction was amended by the DFT-D3 method of Grimme. The Gibbs free energy for intermediates of CO_2 reduction was

calculated as $\Delta G = \Delta E + \Delta E_{ZPE} - T\Delta S$, where the ΔE, ΔE_{ZPE}, and ΔS are electronic energy, zero-point energy, and entropy difference between products and reactants. The zero-point energies of isolated and adsorbed molecules were calculated from the frequency analysis. The entropies of molecules in the gas phase were taken from the National Institute of Standards and Technology (NIST) database.

Supplementary Figures

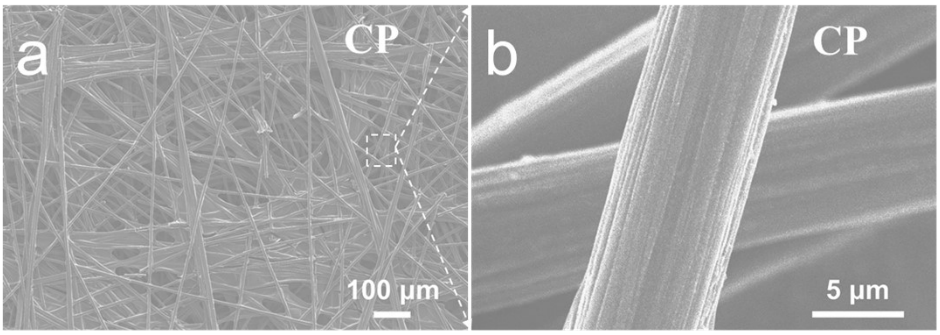

Fig. S1. (a, b) SEM images of CP with different magnifications.

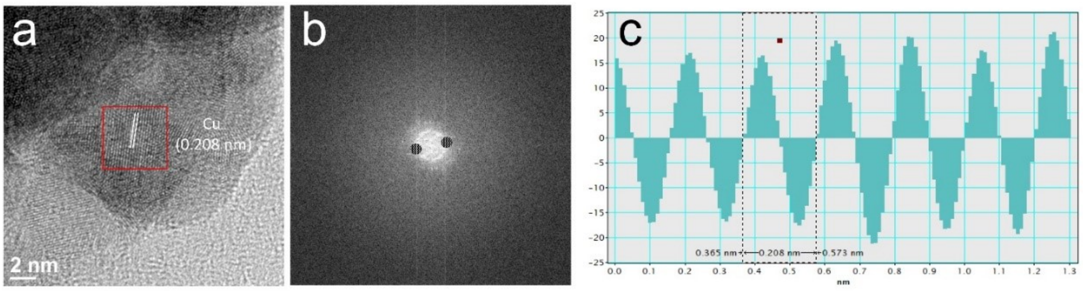

Fig. S2. (a) HR-TEM image of as-synthesized pure Cu metallic film catalyst; (b) Live FFT of as-synthesized pure Cu catalyst with the selected area; (c) Profile of IFFT of as-synthesized pure Cu catalyst with the selected area.

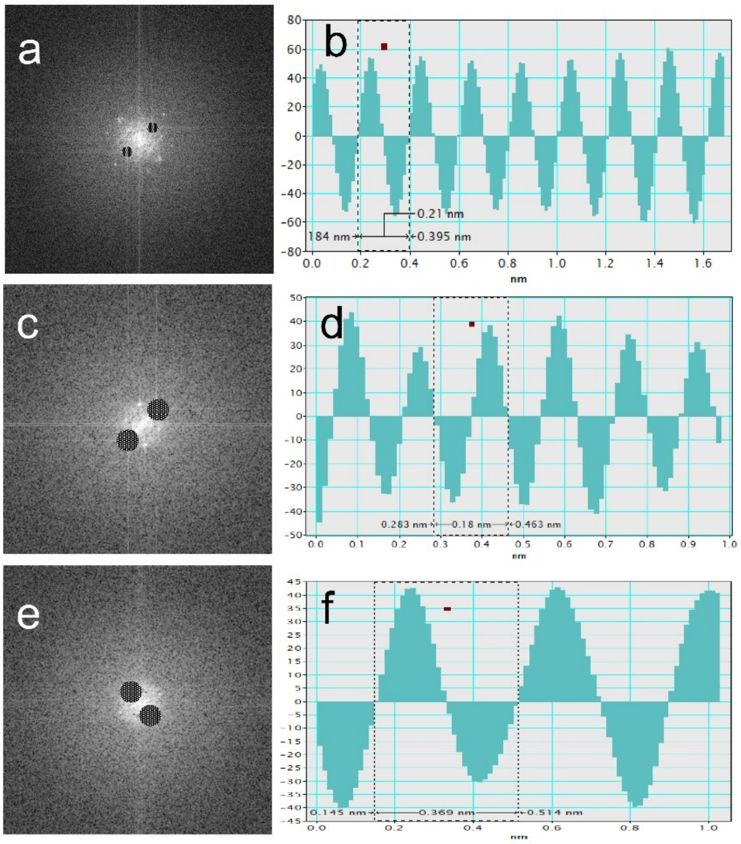

Fig. S3. (a, c, e) Live FFT of the as-synthesized $Cu_{10}La_1Cs_1$ catalyst with the selected area of Cu, La, and Cs; (b, d, f) Profile of IFFT of the as-synthesized $Cu_{10}La_1Cs_1$ catalyst with the selected area of Cu, La, and Cs.

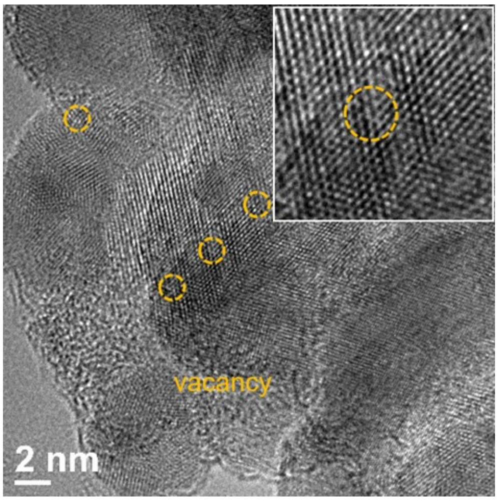

Fig. S4. Defect analysis images of the as-synthesized $Cu_{10}La_1Cs_1$ catalyst through HR-TEM: vacancy (yellow dashed circles).

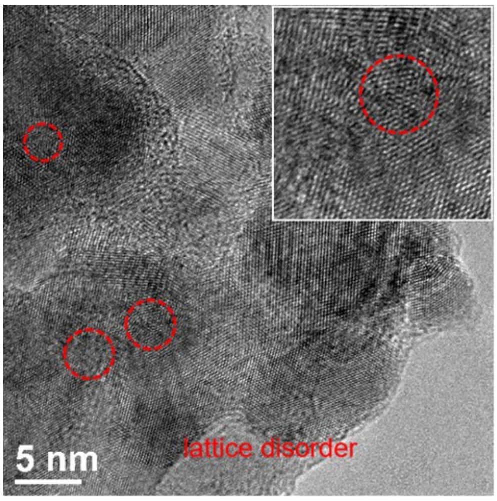

Fig. S5. Defect analysis images of the as-synthesized $Cu_{10}La_1Cs_1$ catalyst through HR-TEM: lattice disorder (red dashed circles).

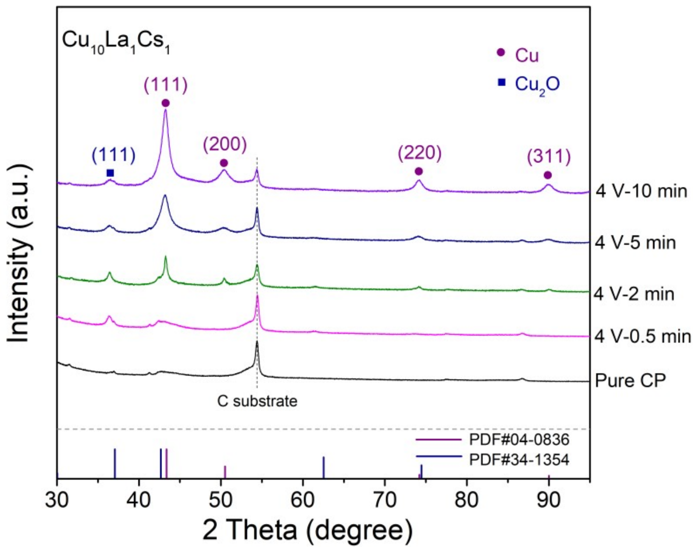

Fig. S6. XRD patterns of virgin CP and $Cu_{10}La_1Cs_1$ catalysts prepared at different electrodeposition time with a constant voltage of 4V.

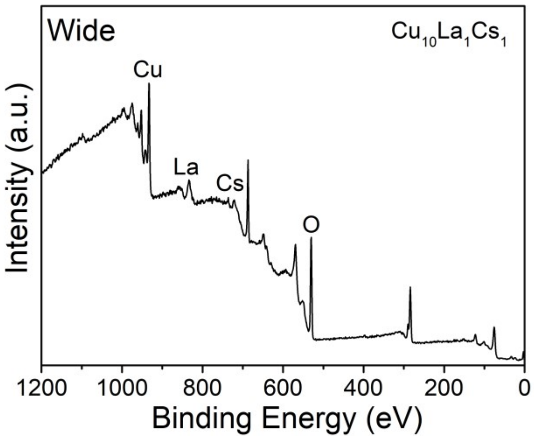

Fig. S7. XPS spectrum of $Cu_{10}La_1Cs_1$ catalyst.

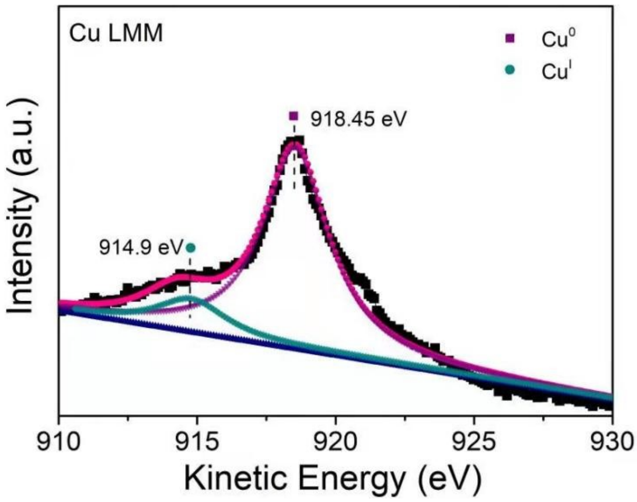

Fig. S8. Auger Cu LMM spectra of $Cu_{10}La_1Cs_1$ catalyst.

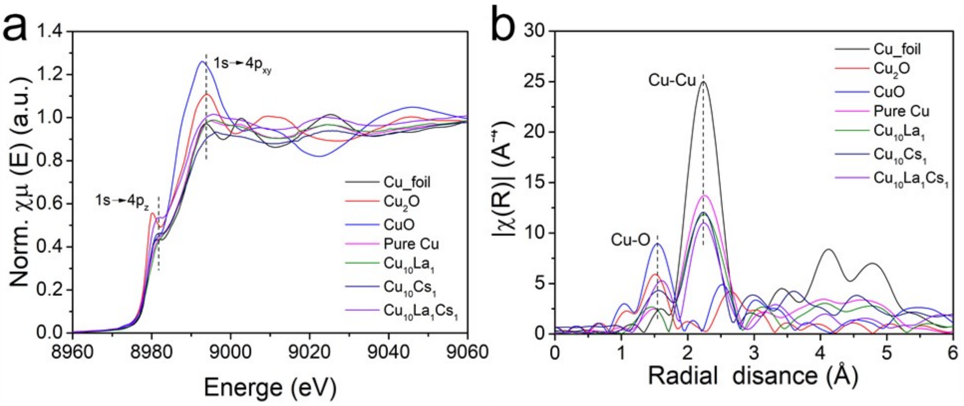

Fig. S9. (a) Normalized Cu Kedge XANES spectra and (b) Corresponding k3-weighted FT-EXAFS spectra of the different catalysts.

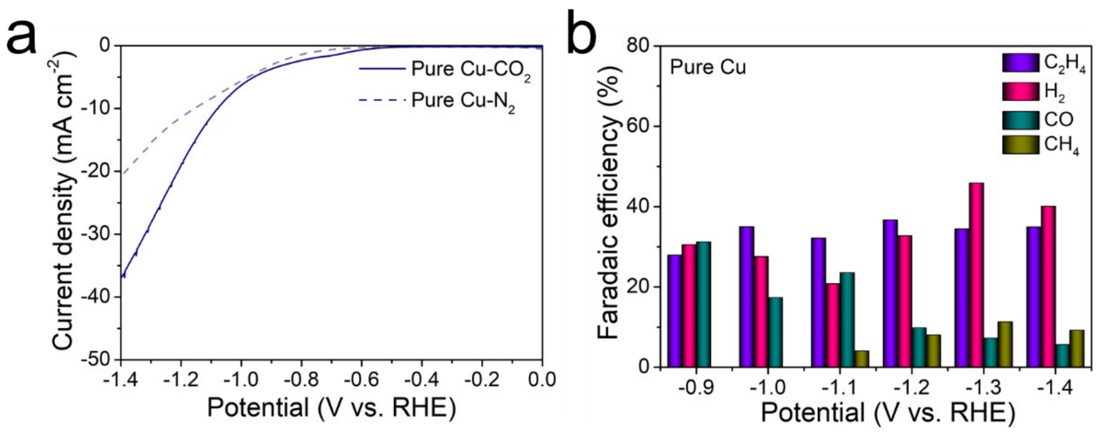

Fig. S10. (a) LSV traces and (b) the distribution of reduction products at different potentials over pure Cu catalyst.

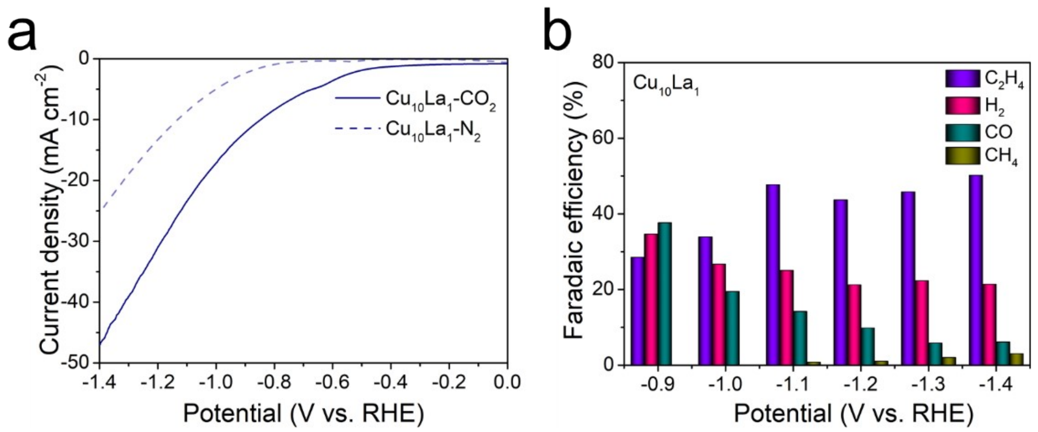

Fig. S11. (a) LSV traces and (b) the distribution of reduction products at different potentials over $Cu_{10}La_1$ catalyst.

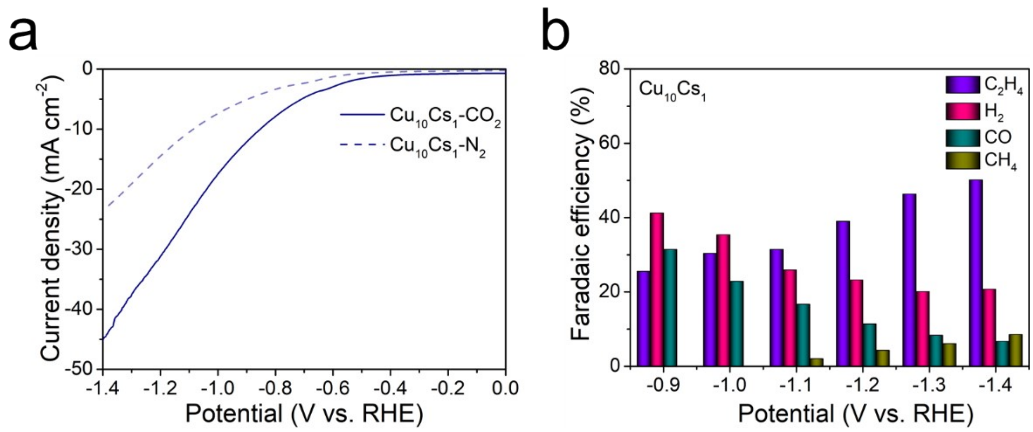

Fig. S12. (a) LSV traces and (b) the distribution of reduction products at different potentials over $Cu_{10}Cs_1$ catalyst.

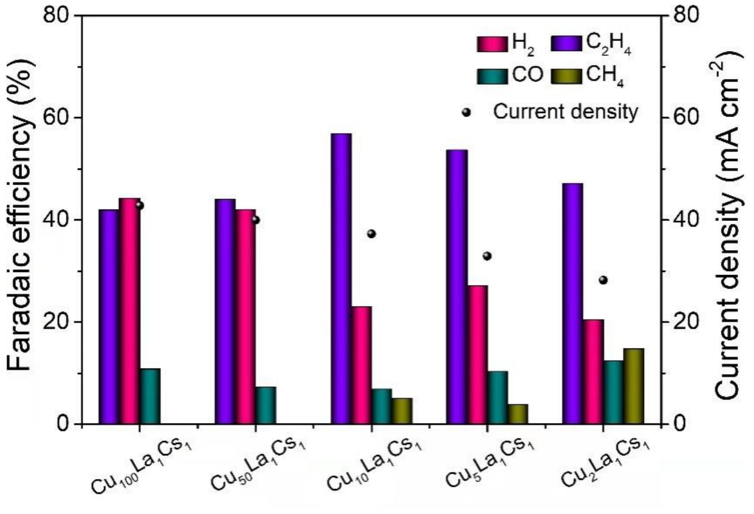

Fig. S13. Distribution of reduction products and current densities over trimetallic catalysts with different Cu-La-Cs ratios.

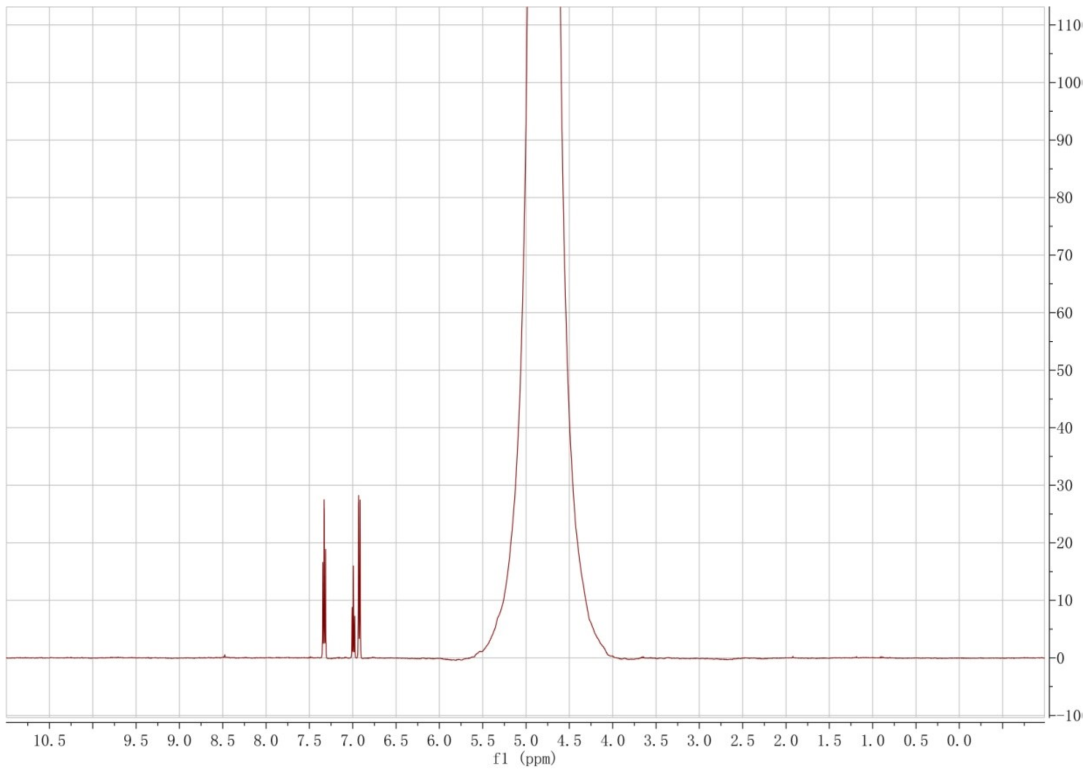

Fig. S14. Represent 1H-NMR spectra of electrolyte after electrolysis on $Cu_{10}La_1Cs_1$ electrode at the potential −1.2 V vs. RHE in 0.1 M KCl aqueous electrolyte using a typical H-type cell. D_2O with phenol was used as an internal standard.

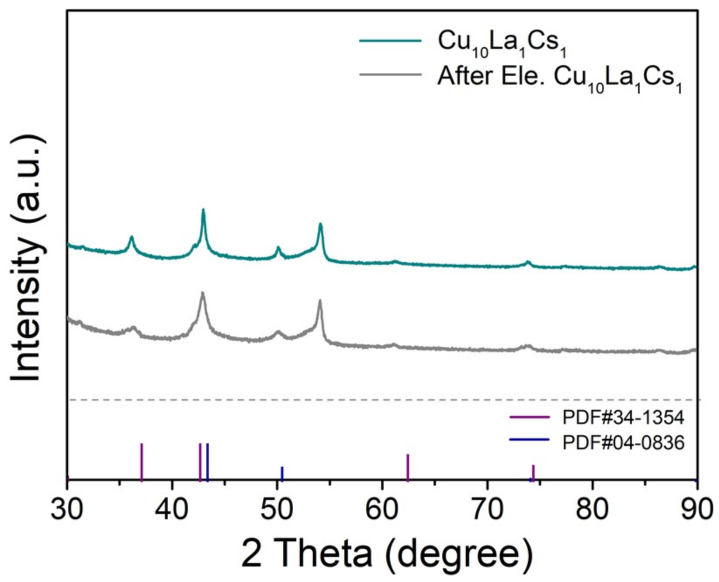

Fig. S15. XRD patterns of $Cu_{10}La_1Cs_1$ catalyst before and after electrolysis.

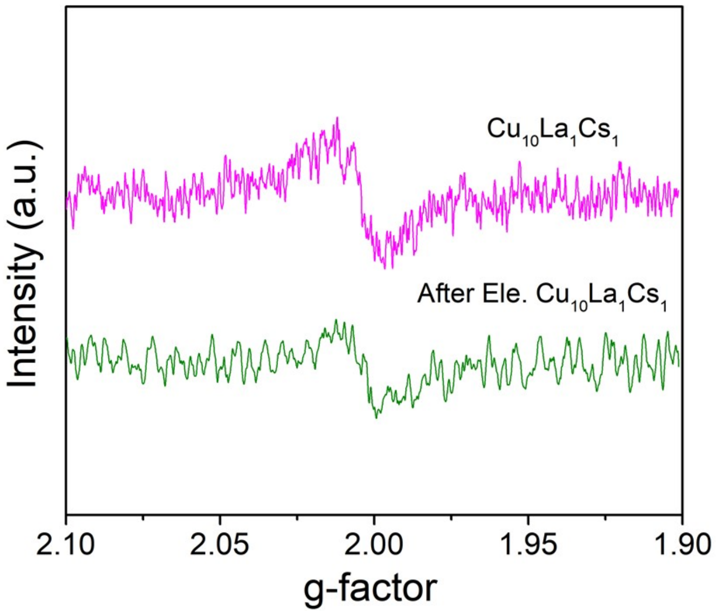

Fig. S16. EPR spectra of $Cu_{10}La_1Cs_1$ catalyst before and after electrolysis.

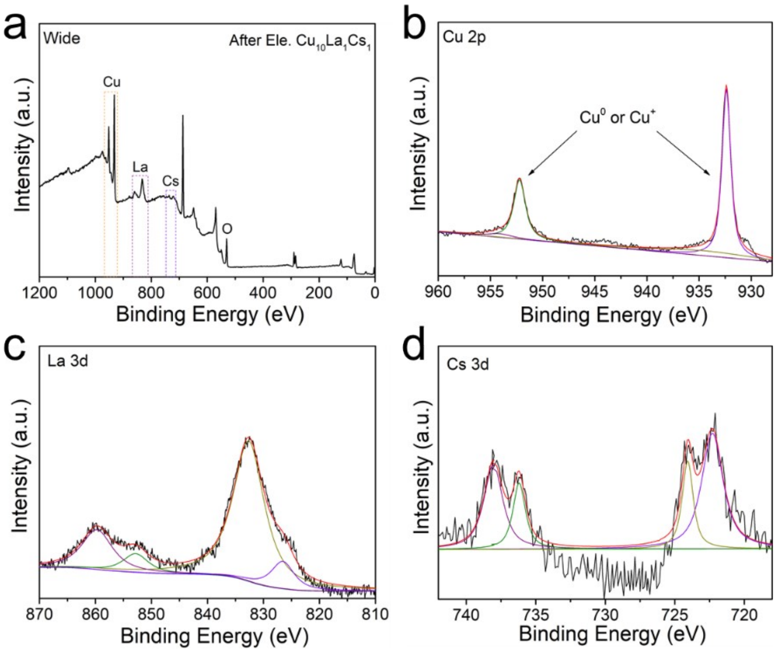

Fig. S17. XPS spectra of (a) wide spectrum, (b) Cu 2p spectra, (c) La 3d spectra, and (d) Cs 3d spectra of $Cu_{10}La_1Cs_1$ catalyst after electrolysis.

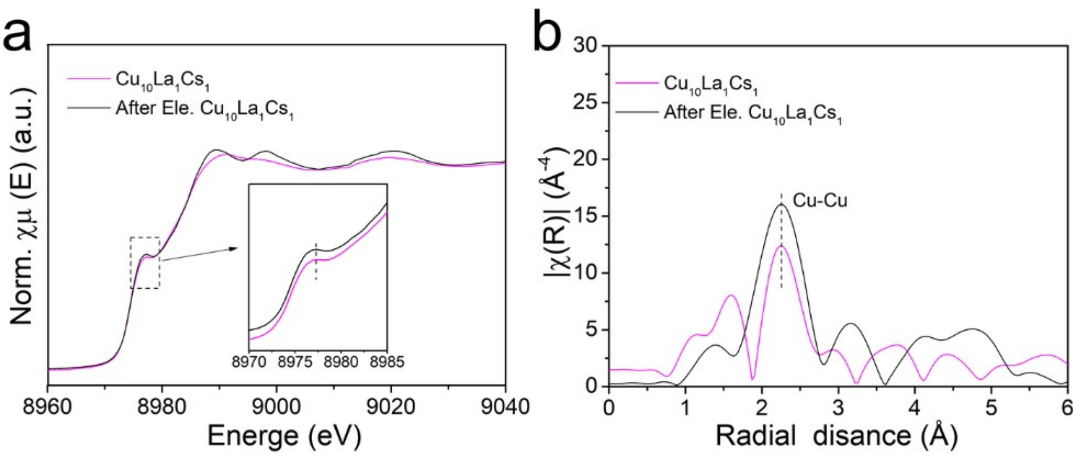

Fig. S18. (a) Normalized Cu Kedge XANES spectra and (b) Corresponding k3-weighted FT-EXAFS spectra of $Cu_{10}La_1Cs_1$ catalyst after electrolysis.

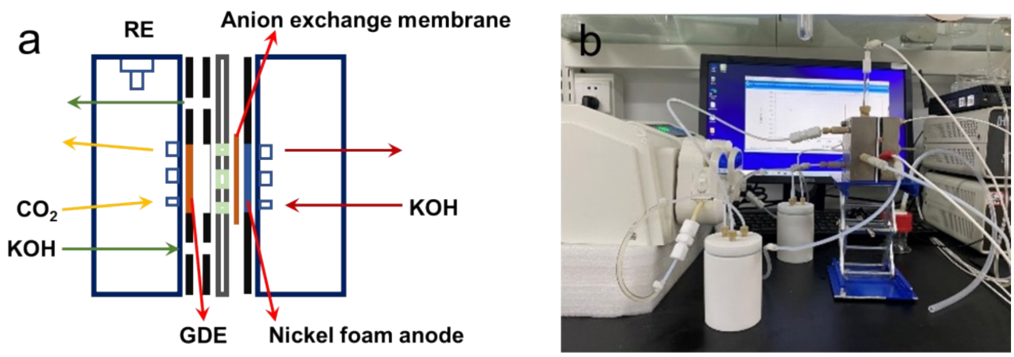

Fig. S19. (a) Schematic and (b) photograph of the flow cell setup used for CO_2RR.

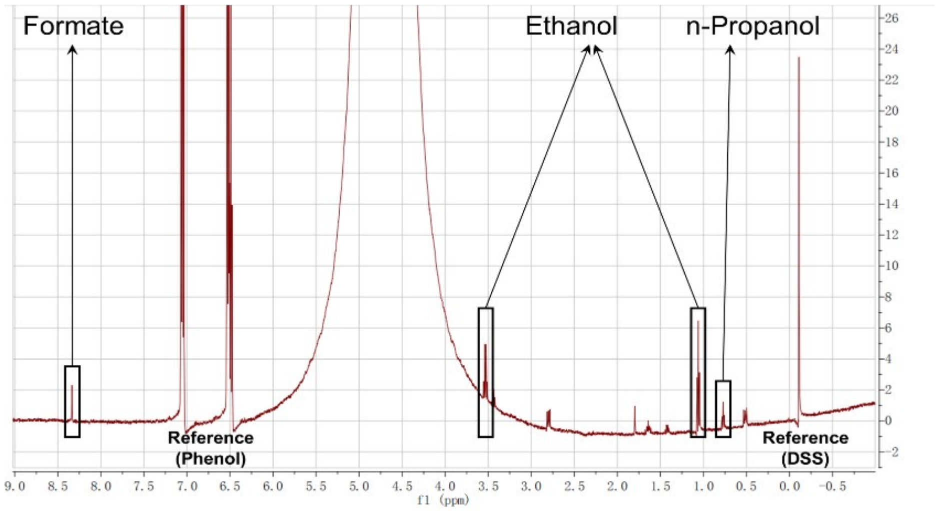

Fig. S20. NMR results of CO_2RR on the trimetallic $Cu_{10}La_1Cs_1$ catalyst in 1 M KOH showing the characteristic peaks of formate, ethanol and n-propanol.

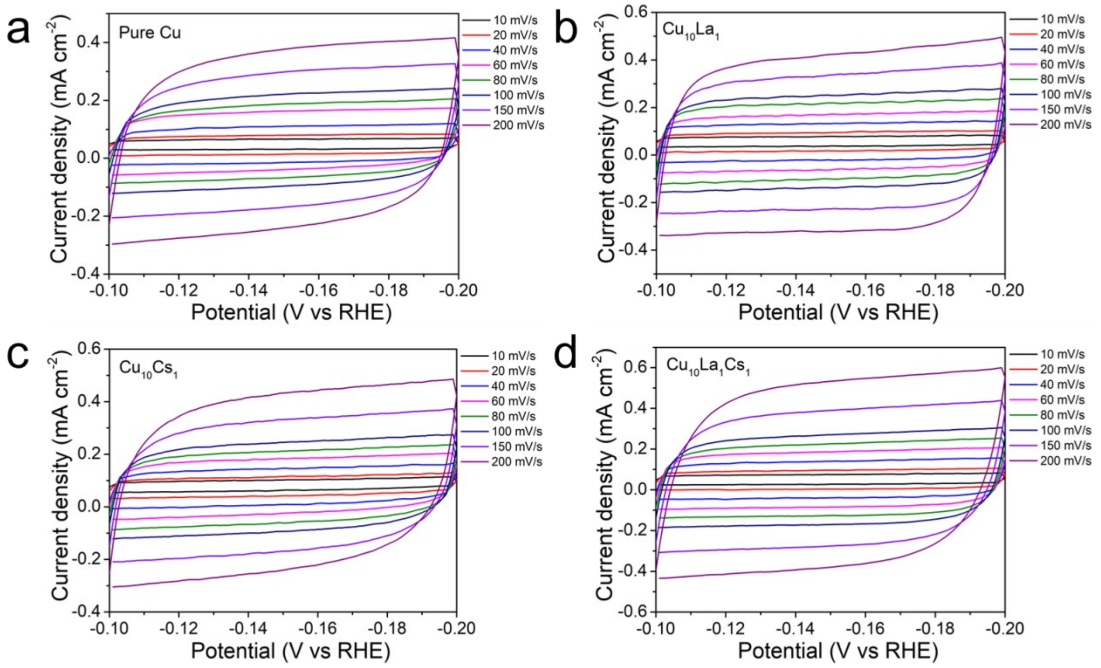

Fig. S21. Determination of ECSA by measuring the C_{dl} from cyclic voltammetries obtained at various scan rates of different catalysts. The ECSA of the working electrodes can be calculated according to the following equation: ECSA = R_fS, where R_f is the roughness factor (defined to be 1) and S is the real surface area of the working electrode (in this work, S = 1 cm^2). The R_f can be calculated by the relation $R_f = C_{dl}/a$, where a is the double-layer capacitance of a smooth Cu surface. Therefore, the ECSA is proportional to C_{dl} value and can be compared via C_{dl} value.

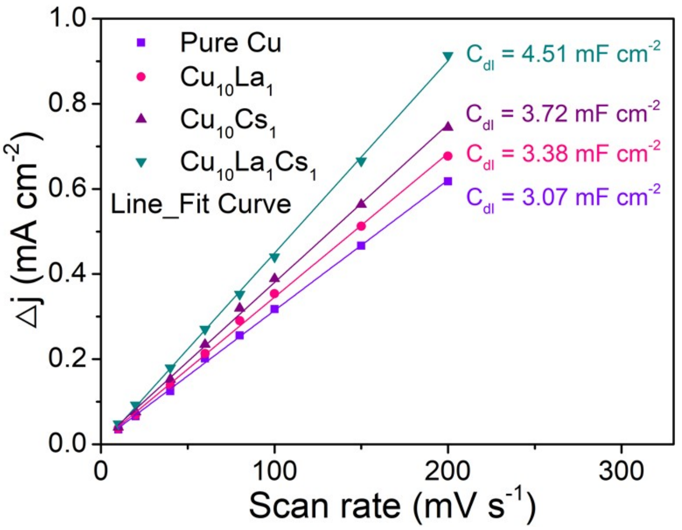

Fig. S22. Charging current density against scan rates over different catalysts in CO_2 saturated 0.1 M KCl aqueous electrolyte.

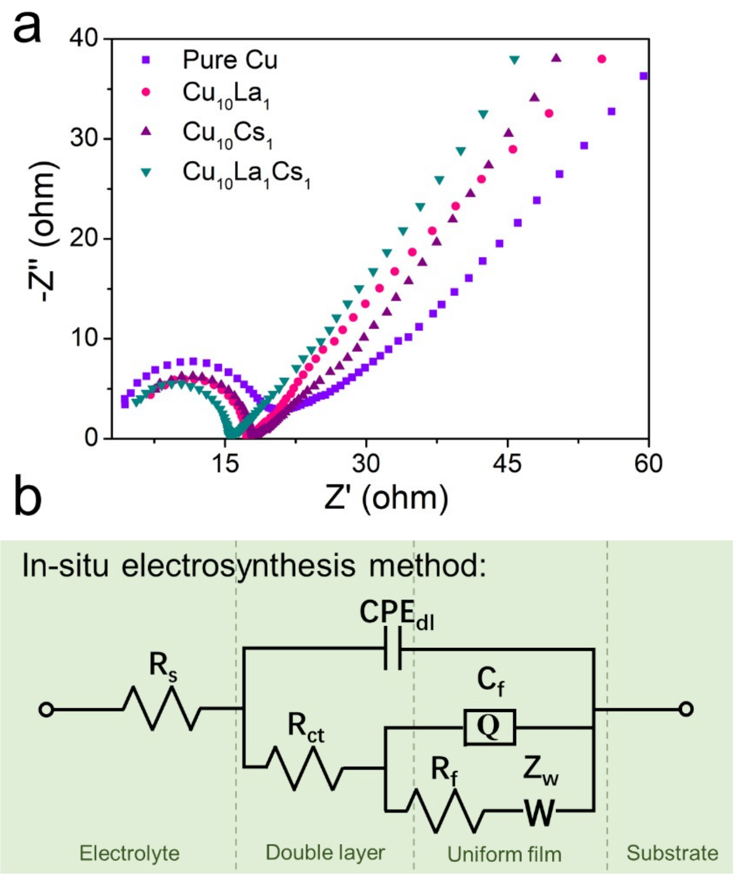

Fig. S23. (a) Nyquist plots of different catalysts in CO_2 saturated 0.1 M KCl solution at OCP; (b) Randles' equivalent circuit used for fitting the experimental impedance data of the electrode prepared by in-situ electrodeposition method: solution resistance (R_s), double layer capacitance (C_{dl}), charge transfer resistance (R_{ct}), film capacitance (C_f), film resistance (R_f) and Warburg-type impedance (Z_w). For in-situ electrodeposition method, the EIS data can be well modeled with a ladder type circuit, which is used to model the uniform film with one or more adsorbed substances.

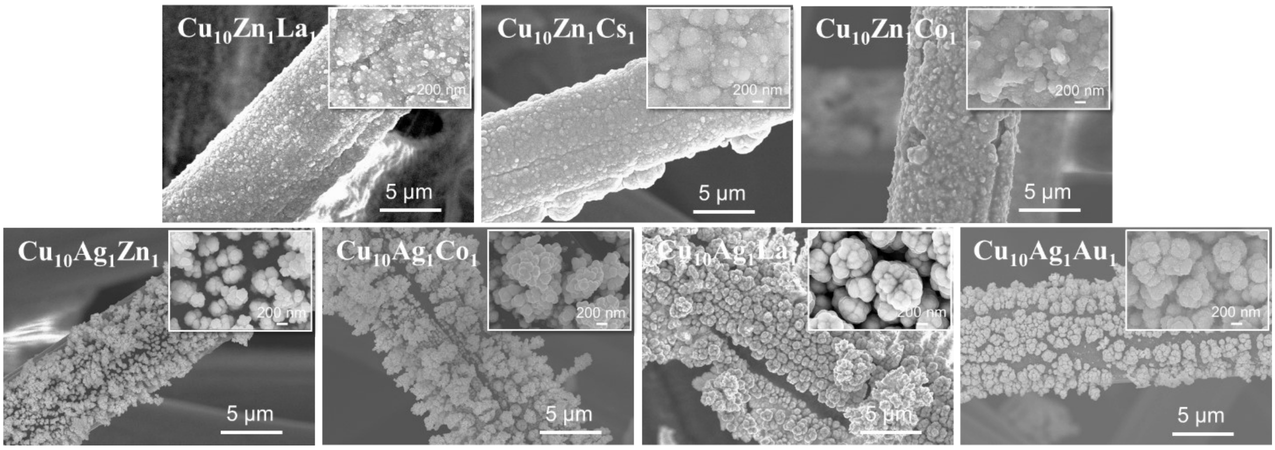

Fig. S24. SEM images and (inset: high-magnification) of trimetallic catalysts (Cu-X-Y) with different metals obtained at a constant voltage of 4 V for 2 min.

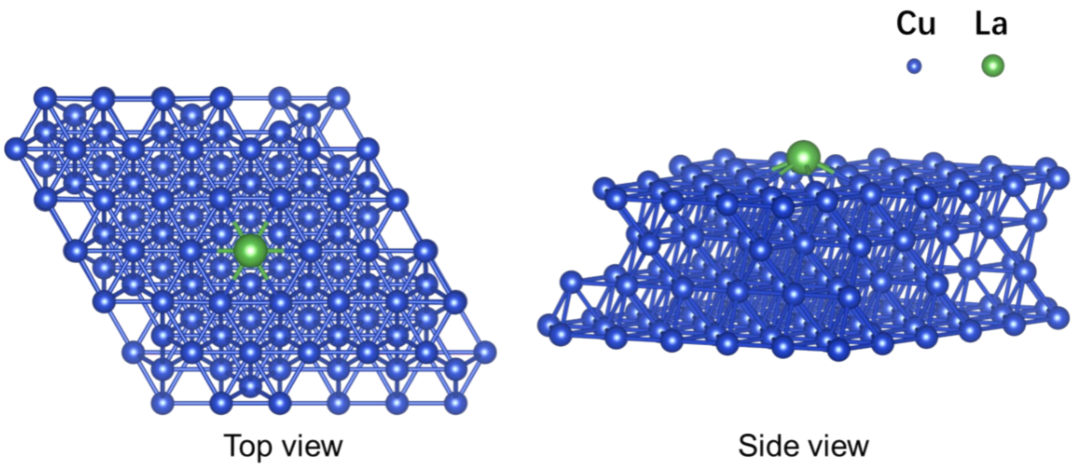

Fig. S25. The top and side views of $Cu_{10}La_1$ structures.

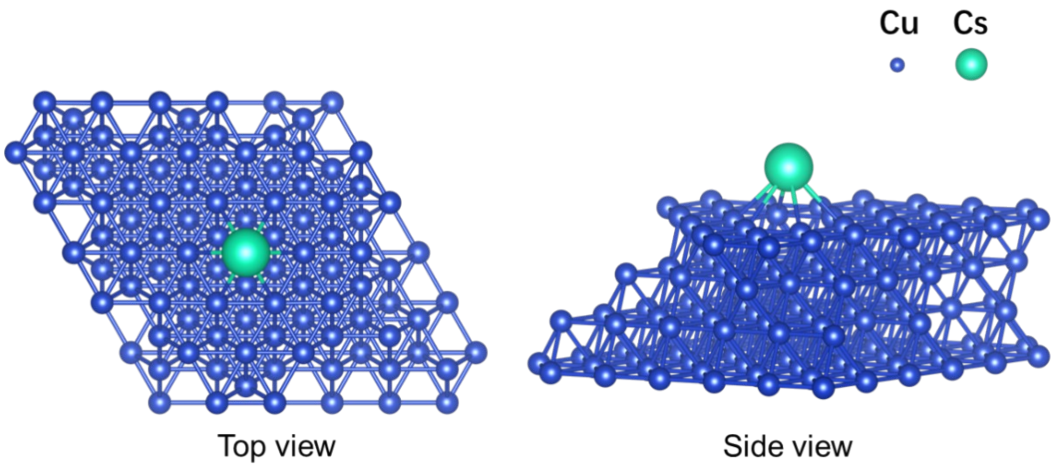

Fig. S26. The top and side views of $Cu_{10}Cs_1$ structures.

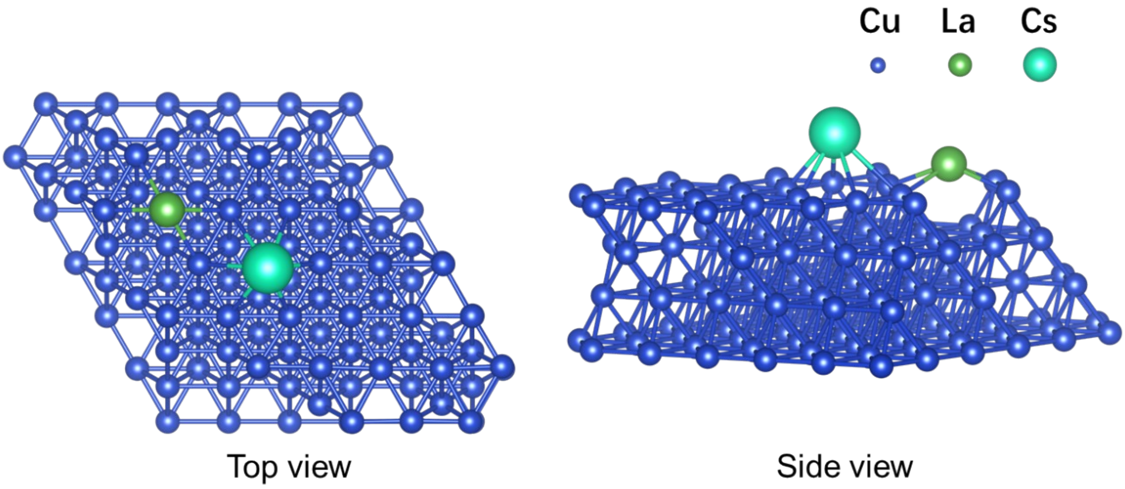

Fig. S27. The top and side views of $Cu_{10}La_1Cs_1$ structures.

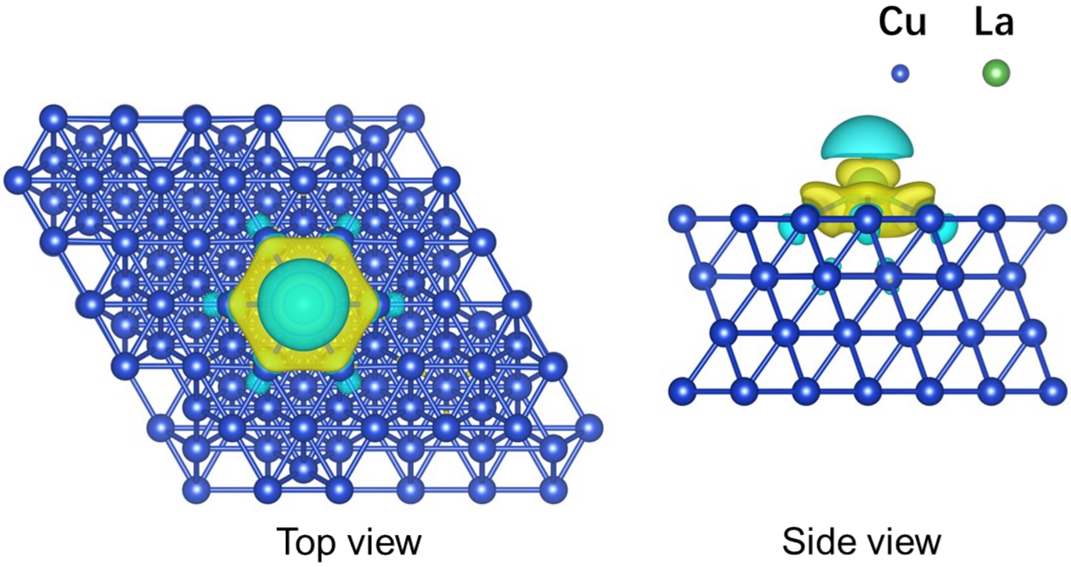

Fig. S28. The top and side views of the charge density difference of $Cu_{10}La_1$ with an isosurface of $2*10^{-3}$ e/$Å^3$. (The charge accumulation is shown as the yellow region, and the charge depletion is shown as the cyan region.).

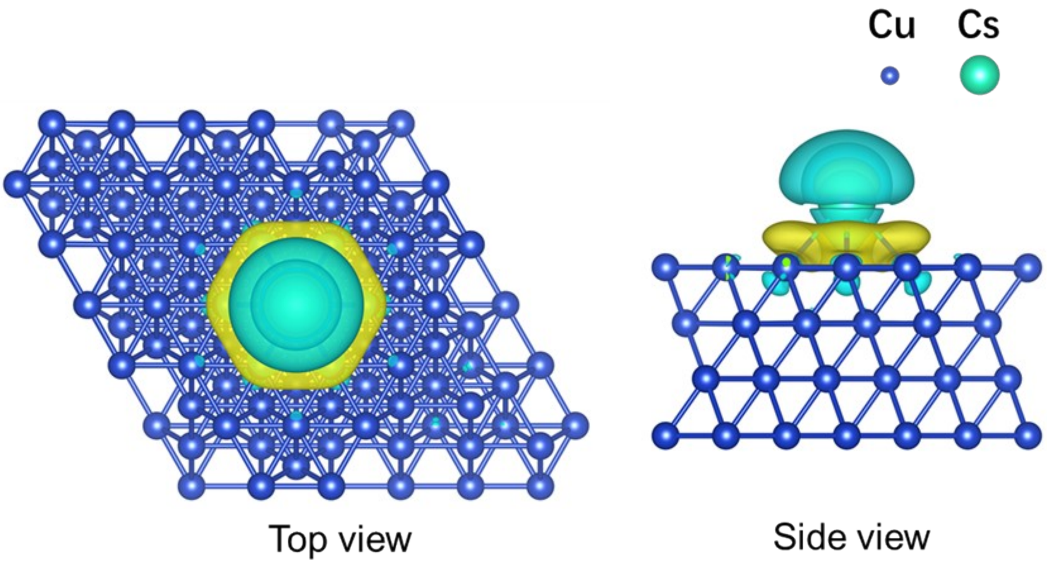

Fig. S29. The top and side views of the charge density difference of $Cu_{10}Cs_1$ with an isosurface of $6*10^{-4}$ e/Å^3. (The charge accumulation is shown as the yellow region, and the charge depletion is shown as the cyan region.).

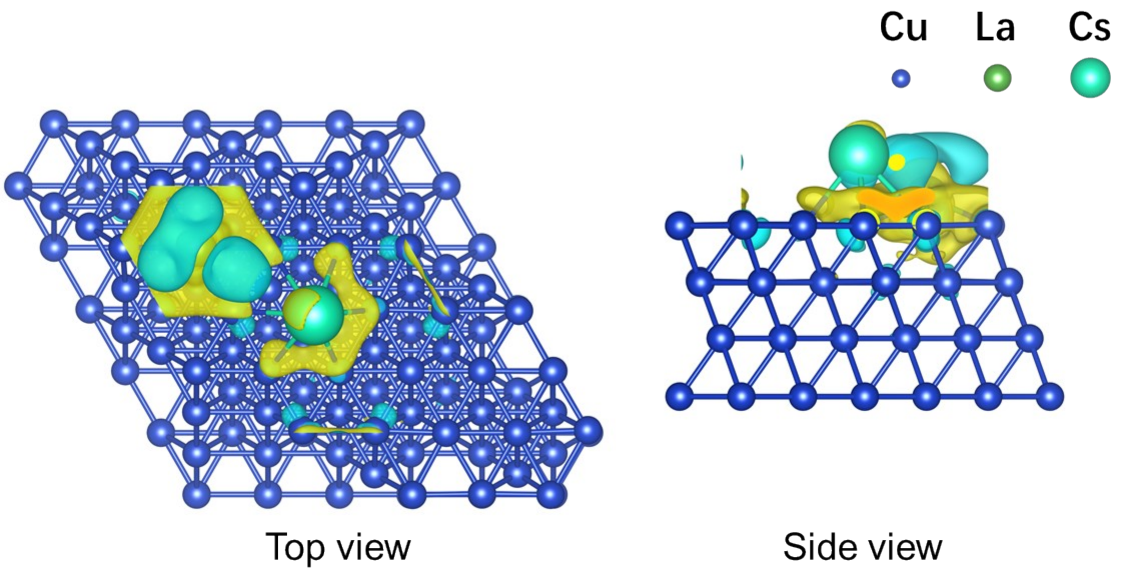

Fig. S30. The top and side views of the charge density difference of $Cu_{10}La_1Cs_1$ with an isosurface of $2*10^{-3}$ e/Å^3. (The charge accumulation is shown as the yellow region, and the charge depletion is shown as the cyan region.).

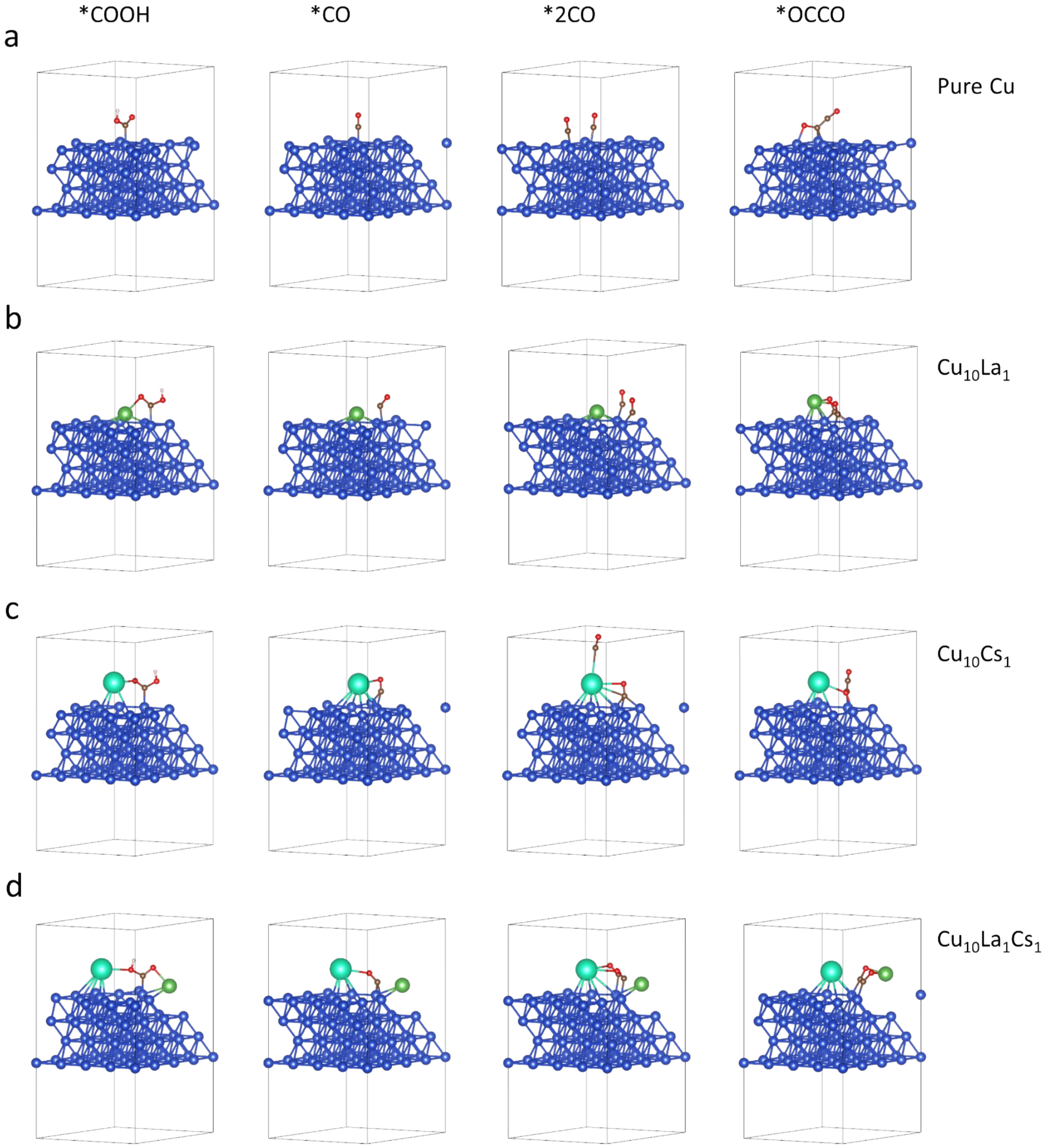

Fig. S31. The optimized adsorption configurations of reaction intermediates on the four simulated interface structures. The atoms in blue, green, viridian, brown, red, and white represent Cu, La, Cs, C, O, and H, respectively.

Supplementary Tables

Table S1. Comparison of C_2H_4 selectivities of trimetallic, bimetallic, and monometallic Cu based catalysts tested in H-type Cell.

Catalysts	Electrolyte	Potential (V vs RHE)	FE (C_2H_4) (%)	j_{C2H4} (mA cm^{-2})	Reference
$Cu_{10}La_1Cs_1$	0.1 M KCl	−1.2	56.9	21.3	*This work*
Cu_3-Ag_3Au nanoframes	0.1 M $KHCO_3$	−1.2	69 ± 5	13.06	6
La_2CuO_4 Perovskite Nanobamboos	0.1 M $KHCO_3$	−1.0	60	3.0	7
Cu-Ag nanowires	0.1 M $KHCO_3$	−1.05	52	18.07	8
Cu-Sb	0.1 M KCl	−1.19	49.7	14.6	9
Cu-Pd catalysts	0.1 M KCl	−1.2	45.2	7.86	10
Au@Cu	0.1 M $KHCO_3$	−1.11	44.9	14.4	11
Ag-Cu arrays	0.5 M $KHCO_3$	−1.2	41.3	8.45	12
Ag_1-$Cu_{1.1}$ nanodimers	0.1 M $KHCO_3$	−1.1	40	1.0	13
CuAu	0.1 M $NaHCO_3$	−1.05	39	10.9	14
Cu-Zn bimetallic catalyst	0.1 M $KHCO_3$	−1.1	33.3	6.1	15
Nanodefective Cu Nanosheets	0.1 M K_2SO_4	−1.18	83.2	48.9	16
p-NG/Cu	0.5 M $KHCO_3$	−0.9	79	7.11	17
Branched CuO nanoparticles	0.1 M $KHCO_3$	−1.05	68±5	20.4	18

nanostructured oxide layer	0.1 M $KHCO_3$	−0.9	60	12.6	19
Cu/PANI-CP	0.1 M KCl	−1.2	59.4	17.94	20
Cu_2O NP/C	0.1 M $KHCO_3$	−1.1	57.3	13.18	21
Hydrophobic Cu dendrite	0.1 M $CsHCO_3$	−1.5	56	16.8	22
Plasmaoxidized Cu	0.1 M $KHCO_3$/ 0.3 M KI	−1.0	47.6	21.65	23
Oxygen-bearing Cu	0.5 M $KHCO_3$	−0.95	45	20.12	24
Cu_2S-Cu-V	0.1 M $KHCO_3$	−1.1	42	12.6	25
44 nm Cu NC cubes	0.1 M $KHCO_3$	−1.1	41	4.5	26
CV-treated Cu	0.1 M $KHCO_3$	−1.0	40	–	27
Anodized Cu	0.1 M $KHCO_3$	−1.08	38.1	7.3	28
Porous Cu	0.1 M $KHCO_3$	−1.0	36.3	7.26	29
Prism-Cu	0.1 M $KHCO_3$	−1.15	30	12	30

Table S2. The average loading mass of $Cu_{10}La_1Cs_1$ metallic film electrocatalysts, measured the CP using a high-precision microbalance before and after the electrodeposition.

Catalysts		1（mg）	2（mg）	3（mg）	Average mass（mg）
$Cu_{10}La_1Cs_1$	Before	8.3	8.7	8.6	8.53
	After	9.7	10.0	9.9	9.86
	Loading mass	1.4	1.3	1.3	1.33

Table S3. Elemental composition in (w/w) of the investigated samples determined by ICP-OES.

Catalysts (Cu-X-Y)	Cu (wt%)	X (wt%)	Y (wt%)
$Cu_5La_1Cs_1$	88.12	5.56	6.31
$Cu_{10}La_1Cs_1$	93.92	3.31	2.77
$Cu_{10}Ag_1La_1$	79.08	16.83	4.09
$Cu_{10}Zn_1La_1$	85.70	9.86	4.44
$Cu_{10}Zn_1Cs_1$	87.45	9.39	3.16
$Cu_{10}Zn_1Co_1$	80.73	9.43	9.84
$Cu_{10}Ag_1Co_1$	72.68	18.30	9.01
$Cu_{10}Ag_1Zn_1$	75.99	15.26	8.75
$Cu_{10}Ag_1Au_1$	69.55	16.34	14.12

Table S4. Elemental composition in (mol/mol) of the investigated samples according to ICP-OES data.

Catalysts (Cu-X-Y)	Cu	X	Y
$Cu_5La_1Cs_1$	5	0.14	0.17
$Cu_{10}La_1Cs_1$	10	0.16	0.14
$Cu_{10}Ag_1La_1$	10	1.26	0.24
$Cu_{10}Zn_1La_1$	10	1.13	0.24
$Cu_{10}Zn_1Cs_1$	10	1.06	0.17
$Cu_{10}Zn_1Co_1$	10	1.15	1.32
$Cu_{10}Ag_1Co_1$	10	1.49	1.34
$Cu_{10}Ag_1Zn_1$	10	1.19	1.13
$Cu_{10}Ag_1Au_1$	10	1.39	0.66

References

1 B. Ravel and M. Newville, *J. Synchrotron. Radiat.*, 2005, **12**, 537-541.

2 G. Kresse and J. Furthmüller, *Physical. Review. B.*, 1996, **54**, 11169-11186.

3 G. Kresse and J. Furthmüller, *Comput. Mater. Sci.*, 1996, **6**, 15-50.

4 J. P. Perdew, K. Burke and M. Ernzerhof, *Phys. Rev. Lett.*, 1996, **77**, 3865-3868.

5 J. P. Perdew, K. Burke and M. Ernzerhof, *Phys. Rev. Lett.*, 1997, **78**, 1396-1396.

6 L. Xiong, X. Zhang, H. Yuan, J. Wang, X. Yuan, Y. Lian, H. Jin, H. Sun, Z. Deng, D. Wang, J. Hu, H. Hu, J. Choi, J. Li, Y. Chen, J. Zhong, J. Guo, M. H. Rummerli, L. Xu and Y. Peng, *Angew. Chem. Int. Ed.*, 2021, **60**, 2508-2518.

7 J. Wang, C. Cheng, B. Huang, J. Cao, L. Li, Q. Shao, L. Zhang and X. Huang, *Nano Lett.*, 2021, **21**, 980-987.

8 J. Gao, H. Zhang, X. Guo, J. Luo, S. M. Zakeeruddin, D. Ren and M. Gratzel, *J. Am. Chem. Soc.*, 2019, **141**, 18704-18714.

9 S. Jia, Q. Zhu, H. Wu, M. e. Chu, S. Han, R. Feng, J. Tu, J. Zhai and B. Han, *Chin. J. Catal.*, 2020, **41**, 1091-1098.

10 R. Feng, Q. Zhu, M. Chu, S. Jia, J. Zhai, H. Wu, P. Wu and B. Han, *Green Chem.*, 2020, **22**, 7560-7565.

11 Y. Chen, Z. Fan, J. Wang, C. Ling, W. Niu, Z. Huang, G. Liu, B. Chen, Z. Lai, X. Liu, B. Li, Y. Zong, L. Gu, J. Wang, X. Wang and H. Zhang, *J. Am. Chem. Soc.*, 2020, **142**, 12760-12766.

12 L. Hou, J. Han, C. Wang, Y. Zhang, Y. Wang, Z. Bai, Y. Gu, Y. Gao and X. Yan, *Inorg. Chem. Front.*, 2020, **7**, 2097-2106.

13 J. Huang, M. Mensi, E. Oveisi, V. Mantella and R. Buonsanti, *J. Am. Chem. Soc.*, 2019, **141**, 2490-2499.

14 J. Gao, D. Ren, X. Guo, S. M. Zakeeruddin and M. Gratzel, *Faraday Discuss.*, 2019, **215**, 282-296.

15 Y. Feng, Z. Li, H. Liu, C. Dong, J. Wang, S. A. Kulinich and X. Du, *Langmuir*, 2018, **34**, 13544-13549.

16 B. Zhang, J. Zhang, M. Hua, Q. Wan, Z. Su, X. Tan, L. Liu, F. Zhang, G. Chen, D. Tan, X. Cheng, B. Han, L. Zheng and G. Mo, *J. Am. Chem. Soc.*, 2020, **142**, 13606-13613.

17 D. D. Zhu, J. L. Liu and S. Z. Qiao, *Adv. Mater.*, 2016, **28**, 3423-3452.

18 J. Kim, W. Choi, J. W. Park, C. Kim, M. Kim and H. Song, *J. Am. Chem. Soc.*, 2019, **141**, 6986-6994.

19 H. Mistry, A. S. Varela, C. S. Bonifacio, I. Zegkinoglou, I. Sinev, Y.-W. Choi, K. Kisslinger, E. A. Stach, J. C. Yang, P. Strasser and B. R. Cuenya, *Nat. Commun.*, 2016, **7**, 12123.

20 S. Jia, Q. Zhu, M. Chu, S. Han, R. Feng, J. Zhai, W. Xia, M. He, H. Wu and B. Han, *Angew. Chem. Int. Ed.*, 2021, **60**, 10977-10982.

21 H. Jung, S. Y. Lee, C. W. Lee, M. K. Cho, D. H. Won, C. Kim, H.-S. Oh, B. K. Min and Y. J. Hwang, *J. Am. Chem. Soc.*, 2019, **141**, 4624-4633.

22 D. Wakerley, S. Lamaison, F. Ozanam, N. Menguy, D. Mercier, P. Marcus, M. Fontecave and V. Mougel, *Nat. Mater.*, 2019, **18**, 1222-1227.

23 D. Gao, F. Scholten and B. Roldan Cuenya, *ACS Catal.*, 2017, **7**, 5112-5120.

24 W. Zhang, C. Huang, Q. Xiao, L. Yu, L. Shuai, P. An, J. Zhang, M. Qiu, Z. Ren and Y. Yu, *J. Am. Chem. Soc.*, 2020, **142**, 11417-11427.

25 T.-T. Zhuang, Z.-Q. Liang, A. Seifitokaldani, Y. Li, P. De Luna, T. Burdyny, F. Che, F. Meng, Y. Min, R. Quintero-Bermudez, C. T. Dinh, Y. Pang, M. Zhong, B. Zhang, J. Li, P.-N. Chen, X.-L. Zheng, H. Liang, W.-N. Ge, B.-J. Ye, D. Sinton, S.-H. Yu and E. H. Sargent, *Nat. Catal.*, 2018, **1**, 421-428.

26 A. Loiudice, P. Lobaccaro, E. A. Kamali, T. Thao, B. H. Huang, J. W. Ager and R. Buonsanti, *Angew. Chem. Int. Ed.*, 2016, **55**, 5789-5792.

27 T. C. Chou, C. C. Chang, H. L. Yu, W. Y. Yu, C. L. Dong, J. J. Velasco-Velez, C. H. Chuang, L. C. Chen, J. F. Lee, J. M. Chen and H. L. Wu, *J. Am. Chem. Soc.*, 2020, **142**, 2857-2867.

28 S. Y. Lee, H. Jung, N. K. Kim, H. S. Oh, B. K. Min and Y. J. Hwang, *J. Am. Chem. Soc.*, 2018, **140**, 8681-8689.

29 M. Li, Y. Ma, J. Chen, R. Lawrence, W. Luo, M. Sacchi, W. Jiang and J. Yang, *Angew. Chem. Int. Ed.*, 2021, **60**, 11487-11493.

30 H. S. Jeon, S. Kunze, F. Scholten and B. Roldan Cuenya, *ACS Catal.*, 2017, **8**, 531-535.
